# Real-time imaging of RNA polymerase I activity in living human cells

**DOI:** 10.1083/jcb.202202110

**Published:** 2022-10-25

**Authors:** Yujuan Fu, Yaxin Liu, Tanye Wen, Jie Fang, Yalong Chen, Ziying Zhou, Xinyi Gu, Hao Wu, Jinghao Sheng, Zhengping Xu, Wei Zou, Baohui Chen

**Affiliations:** 1 Department of Cell Biology and Bone Marrow Transplantation Center of the First Affiliated Hospital, Zhejiang University School of Medicine, Hangzhou, China; 2 Liangzhu Laboratory, Zhejiang University Medical Center, Hangzhou, China; 3 Institute of Environmental Medicine, and Department of General Surgery, Sir Run Run Shaw Hospital, Zhejiang University School of Medicine, Hangzhou, China; 4 The Fourth Affiliated Hospital, Zhejiang University School of Medicine, Yiwu, China; 5 Insititute of Translational Medicine, Zhejiang University, Hangzhou, China; 6 Institute of Hematology, Zhejiang University and Zhejiang Engineering Laboratory for Stem Cell and Immunotherapy, Hangzhou, China; 7 Zhejiang Provincial Key Laboratory of Genetic & Developmental Disorders, Hangzhou, China

## Abstract

RNA polymerase I (Pol I) synthesizes about 60% of cellular RNA by transcribing multiple copies of the ribosomal RNA gene (rDNA). The transcriptional activity of Pol I controls the level of ribosome biogenesis and cell growth. However, there is currently a lack of methods for monitoring Pol I activity in real time. Here, we develop LiveArt (live imaging-based analysis of rDNA transcription) to visualize and quantify the spatiotemporal dynamics of endogenous ribosomal RNA (rRNA) synthesis. LiveArt reveals mitotic silencing and reactivation of rDNA transcription, as well as the transcriptional kinetics of interphase rDNA. Using LiveArt, we identify SRFBP1 as a potential regulator of rRNA synthesis. We show that rDNA transcription occurs in bursts and can be altered by modulating burst duration and amplitude. Importantly, LiveArt is highly effective in the screening application for anticancer drugs targeting Pol I transcription. These approaches pave the way for a deeper understanding of the mechanisms underlying nucleolar functions.

## Teaser

Fu et al. develop a new tool for real-time monitoring of ribosomal RNA synthesis in living cells. This technology can be used to quantitatively measure RNA polymerase I (Pol I) transcriptional activity under various conditions, which facilitates large-scale screening of Pol I regulators.

## Introduction

Precise regulation of ribosome biogenesis is a crucial cellular process, which has the overwhelming burden of efficiently and accurately producing all proteins in the cell. The rate-limiting step in building ribosomes is the synthesis of ribosomal RNA (rRNA) by RNA polymerase I (Pol I; Grummt, 2003; McStay and Grummt, 2008; Moss and Stefanovsky, 2002). The nucleolus is formed around transcribed rRNA genes (rDNA), which are organized as tandem repeats on different chromosomes (Stults et al., 2008). Each transcriptional active rDNA encodes a long 47S pre-rRNA transcript that is processed and modified to generate 18S, 5.8S, and 28S rRNAs. These events occur within distinct nucleolar subcompartments, including fibrillar center (FC), dense fibrillar component (DFC), and granular component (GC; Scheer and Hock, 1999; Thiry and Lafontaine, 2005).

Proper cellular control of rRNA synthesis influences proliferation and differentiation during development (Poortinga et al., 2011; Savic et al., 2014; Woolnough et al., 2016; Zhang et al., 2014a). Moreover, the decline of rRNA synthesis during aging is a risk factor for neurodegenerative diseases (Parlato and Kreiner, 2013). In contrast, an increase in rRNA synthesis actively contributes to cancer progression. This finding has opened up new cancer therapeutic avenues, emphasizing on selective Pol I transcription inhibitors (Bywater et al., 2012; Drygin et al., 2010; Ferreira et al., 2020; Hein et al., 2017; Hein et al., 2013). Targeting Pol I transcription has two major benefits for therapy: (1) Pol I only transcribes 47S rRNA, which has the potential to avoid side effects; (2) Pol I transcription is deregulated in most cancers and therefore its inhibitors have the potential to treat a wide range of cancers (Ferreira et al., 2020). The collective pre-clinical data on selective Pol I transcription inhibitors developed so far illustrate their therapeutic potential for cancer treatment (Khot et al., 2019).

Although dysregulation of rRNA synthesis is closely linked to development and a broad range of human diseases (Hannan et al., 2013; Narla and Ebert, 2010), the spatiotemporal dynamics of rRNA production in living cells remain poorly elucidated. The widespread techniques of Northern blot analysis and RT-PCR measure bulk rRNA levels from homogenous population extracts. Most single-cell detection methods relied on FISH or bromouridine 5′-triphosphate (BrUTP) labeling. The FISH probe recognizing 5′ external transcribed spacer (5′-ETS) or internal transcribed spacer 1 (ITS1) in pre-rRNA has been widely applied to detect pre-rRNA expression (Dundr et al., 2002; Falahati et al., 2016; O'Reilly et al., 1994; Xing et al., 2017; Yao et al., 2019). BrUTP labeling involves the incorporation of modified ribonucleotide analog into the nascent rRNA, followed by immunodetection of BrUTP using a specific antibody (Jackson et al., 1993; Leung et al., 2004; Thiry et al., 2001; Zhang et al., 2014a). However, both FISH and BrUTP labeling methods require fixation of cell samples, which is not suitable for live-cell detection. Other fluorescent probes, such as two-photon fluorescent rRNA probe (J1) and exciton-controlled hybridization-sensitive fluorescent oligonucleotide (ECHO), have been implemented to image intracellular rRNA in living cells (Cao et al., 2019; Du et al., 2017; Oomoto et al., 2015). However, these probes stain rRNAs produced by all rRNA genes and only function in the cells transiently, making it challenging to act as a real-time quantitative labeling system.

Herein, we report a novel rRNA labeling method, LiveArt, which can serve as a real-time reporter of Pol I transcriptional activity in living cells. We applied the MS2/MS2 coat protein (MS2/MCP) system to rRNA tagging. The specificity of rRNA labeling was validated by FISH and the specific Pol I inhibitor. The fluorescent signal of MS2-tagged rRNAs can be dynamically regulated by Pol I regulators as expected. Notably, we were able to isolate clonal cell lines for achieving stable rRNA tagging, which is of utmost importance for long-term live-cell tracking of Pol transcription and large-scale screening of Pol I regulators.

## Results

### Development of LiveArt to image rRNA synthesis in real time

**Figure 1.**
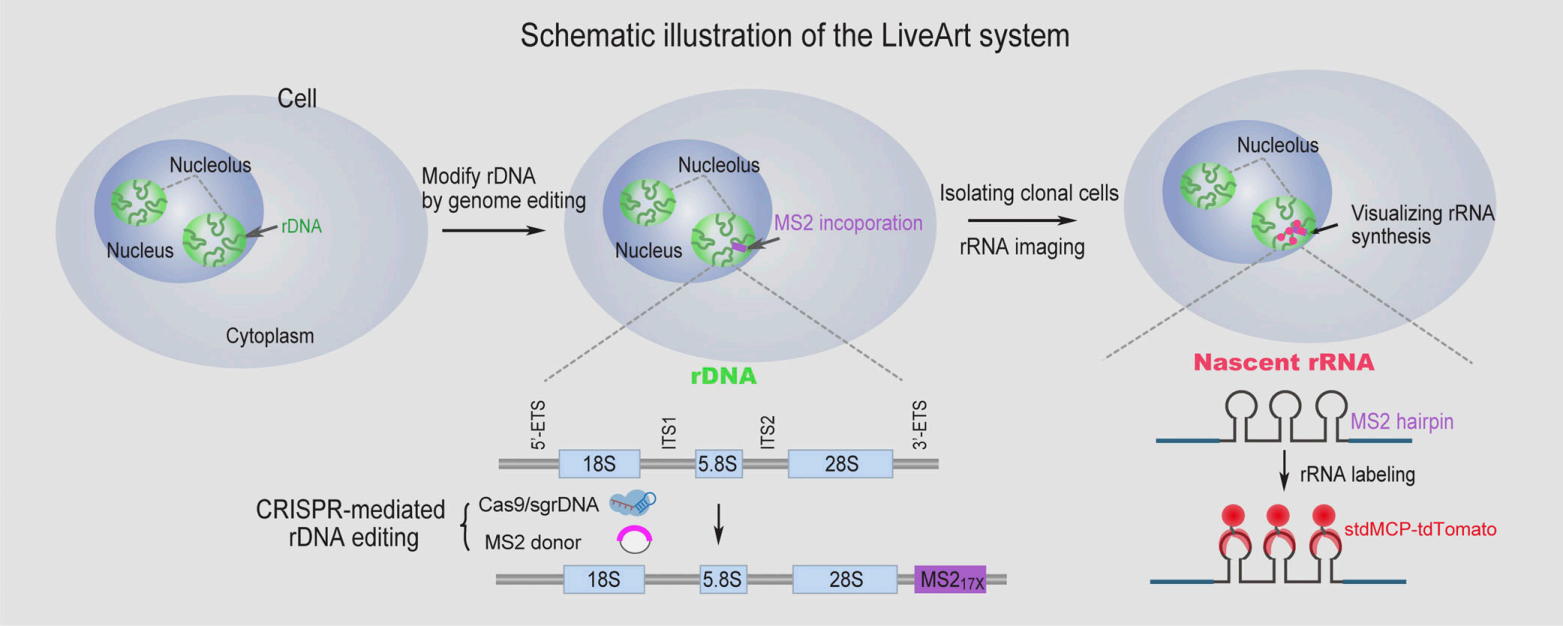
**Principle of LiveArt.** Schematic illustration of LiveArt. 17 repeats of MS2 sequence were knocked into the rDNA transcribed region by CRISPR-Cas9 mediated HITI. Newly synthesized rRNA harboring MS2 hairpins are bound by stdMCP-tdTomato, enabling direct imaging of nascent rRNAs in living cells.

The MS2/MCP system is a widely used method for RNA imaging in living cells (Bertrand et al., 1998; Buxbaum et al., 2015; Das et al., 2021). We envisioned that if MS2 sequence could be incorporated into the transcribed region of rDNA using the genome-editing tool, MS2 hairpins would be transcribed as a part of pre-rRNA. This, combined with exogeneously expressed fluorescently tagged MCP molecules in the cell, would permit visualization of the production of pre-rRNAs, possibly even the spliced rRNAs (18S, 5.8S, and 28S; Fig. 1). To test this hypothesis, we integrated a DNA fragment harboring 17 copies of the MS2 RNA hairpins (MS2V5_17X_) into different regions of rDNA, respectively, through CRISPR-Cas9 mediated homology-independent targeted integration (HITI; Suzuki et al., 2016). These genome editing experiments were performed in HeLa cells stably expressing stdMCP-tdTomato or tdMCP-GFP. Therefore, once the event of MS2 integration and rDNA transcription occur, MS2 tagged pre-rRNA molecules would be bound by stdMCP-tdTomato, thus enabling the visualization of rRNA synthesis.

**Figure S1.**
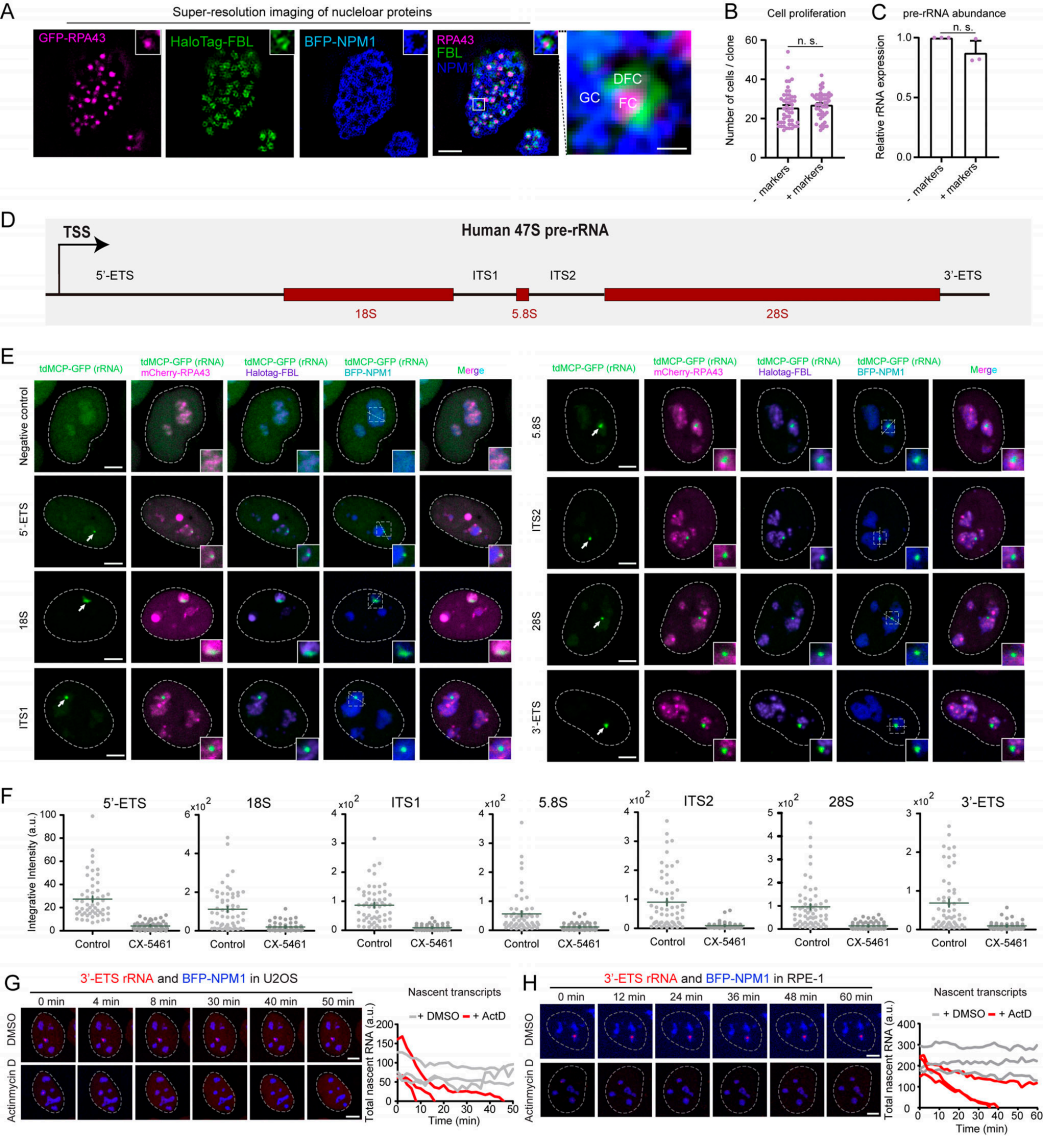
**Imaging rRNAs via tagging various regions in rRNA. (A)** Representative SIM images of nucleoli in living HeLa cells. FC: GFP-RPA43, DFC: HaloTag-FBL, GC: BFP-NPM1. Scale bars, 2 μm and 250 nm (right, enlarged image). Insert magnification: 6×. **(B)** Quantification of the number of cells per clone in cell lines without or with overexpression of three nucleolar markers (RPA43, FBL, and NPM1). Each dot represents a single clone (*n* ≥ 50). **(C)** Measurement of 45S pre-rRNA abundance by qPCR. *n* = 3 biological replicates. Results in B and C are shown as mean ± SEM. Two-tailed unpaired *t* test was analyzed for B and C. n.s., not significant. **(D)** Schematic diagram of human 47S pre-rRNA. TSS, transcription start site. rRNAs can be labeled by integrating MS2 sequence into various regions in rDNA, including 5′-ETS, 18S, ITS1, 5.8S, ITS2, 28S, and 3′-ETS. **(E)** Representative images to show the colocalization of various MS2-tagged rRNAs with standard nucleolar markers in HeLa cells. FC: RPA43, DFC: FBL, GC: NPM1. All the images are maximum-intensity projections from *z* stacks. Scale bars, 5 µm. Insert magnification: 3×. Arrows point to visible MS2-tagged rRNAs. **(F)** Quantification of rRNA accumulation indicated by stdMCP-tdTomato signal intensity in the absence or presence of CX-5461. Each dot represents a single cell (*n* = 100 for all samples). Green line indicates mean ± SEM. **(G and H)** Left: live-cell imaging snapshots of U2OS (G) or RPE-1 (H) cells showing accumulation of MS2-tagged 3′-ETS rRNA indicated by stdMCP-tdTomato in the absence or presence of ActD. BFP-NPM1 was imaged to reveal the nucleoli. All images are maximum-intensity projections from *z* stacks. Scale bars, 5 µm. Right: quantitative analysis of three representative cells showing the dynamic change of 3′-ETS rRNA defined by the total intensity of stdMCP-tdTomato spots in the absence or presence of ActD.

To validate the labeling of MS2-tagged rRNAs, we first constructed three nucleolar markers based on previous studies, including GFP-RPA43 (RNA polymerase I subunit A43 for FC), HaloTag-FBL (Fibrillarin for DFC), and BFP-NPM1 (Nucleophosmin1 for GC; Chen et al., 2005; Dundr et al., 2002; Frottin et al., 2019; Leung et al., 2004; Smirnov et al., 2014; Yao et al., 2019). Using live-cell Hessian structured illumination microscopy (Hessian-SIM), we found that these three fluorescent reporters displayed classic nucleolar architecture as previously observed (Yao et al., 2019). FCs were surrounded by DFCs, while FCs and DFCs were embedded in GCs (Fig. S1 A). Next, we confirmed that the cells overexpressing tagged nucleolar components showed normal rDNA transcriptional activity and cell proliferation (Fig. S1, B and C).

We chose the following regions of rDNA for modification with MS2 insertion: (1) external transcribed spacers (5′-ETS and 3′-ETS), (2) internal transcribed spacers (ITS1 and ITS2), and (3) spliced rRNA products (18S, 5.8S, and 28S; Fig. S1 D). Of note, sgRNA should avoid targeting rRNA processing sites. As expected, enriched tdMCP-GFP foci did appear in some nucleoli in all tagging conditions (Fig. S1 E). We then performed CX-5461 (a specific Pol I inhibitor) treatment to validate the specificity of rRNA labeling. Quantitative analysis showed that MS2-tagged rRNA signals were significantly reduced following CX-5461 treatment. Any of the rRNA labeling strategies exhibited the same results, demonstrating the high specificity of rRNA labeling (Fig. S1 F). In addition to HeLa cells, we also tested rRNA tagging in other cell types, including human nontransformed (RPE-1) and cancer (U2OS) cells. Similarly, fluorescent labeled 3′-ETS rRNAs appeared in the nucleoli and were significantly reduced following Pol I inhibition. Therefore, our results suggest that our rRNA tagging strategy works in multiple cell types (Fig. S1, G and H).

### Stable fluorescent tagging of nascent and mature rRNAs

**Figure S2.**
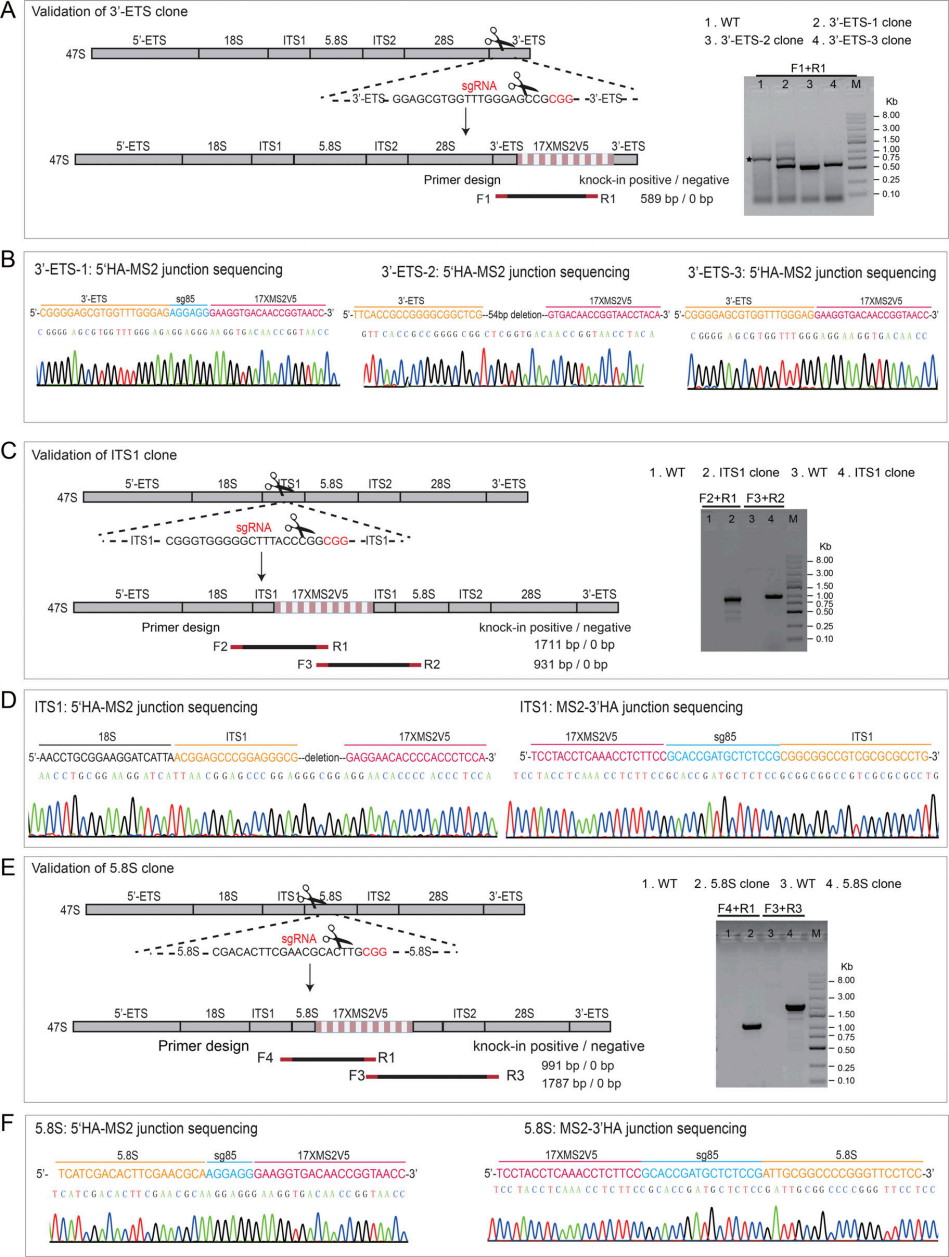
**Validation of CRISPR-based MS2 knockin in the clonal cells. (A, C, and E)** Schematic diagram of primer designs to validate the insertion of MS2V5_17×_ into rDNA at the regions of 3′-ETS (A, three independent clones), ITS1 (C, one clone), and 5.8S (E, one clone) in clonal cells, respectively. sgRNA design for CRISPR knockin is also highlighted to illustrate the insertion site for each region. Representative gels of PCR products are shown to indicate the correct insertions. An unspecific band amplified from 3′-ETS clone is pointed out by asterisk (A, right). The size of all PCR products is correct except the fragment amplified from 3′-ETS clone 2 or ITS1 clone (using F3 and R2 primers), which is shorter than expected due to a deletion in this region revealed by DNA sequencing. Notably, MS2V5-3′HA junction could not be successfully amplified, which might be due to the high GC content (∼80%) in 3′-ETS. **(B, D, and F)** Example chromatogram showing successful recombination for each insertion site at 3′-ETS (B), ITS1 (D), and 5.8S (F). The junction between homology arm and MS2 sequence was shown to indicate the correct insertion. Source data are available for this figure: SourceData FS2.

To achieve stable tagging of rRNAs, we isolated LiveArt clonal cells. Five clonal cell lines were successfully created which will hereafter be referred to as ITS1, 5.8S, 3′-ETS-1, 3′-ETS-2, and 3′-ETS-3 clones. We next validated MS2 insertion by DNA sequencing. Because human rDNA is a highly GC-rich gene (van Sluis and McStay, 2019), it is challenging to amplify rDNA fragments at certain regions for DNA sequencing. By sequencing at least one side of the MS2-rDNA junctions, we found that the MS2 cassette was inserted into the designed position in all five clones (Fig. S2). Deletions in the 5′ HA-MS2 junction were detected in ITS1 and 3′-ETS-2 clones. However, according to the results of our subsequent experiments, this should not affect the determination of rDNA transcription levels. In addition to HITI, CRISPR-mediated homology-directed repair (HDR) can be applied to guide MS2 insertion more precisely (Ran et al., 2013). 3′-ETS-3 clone was isolated using the HDR strategy.

**Figure S3.**
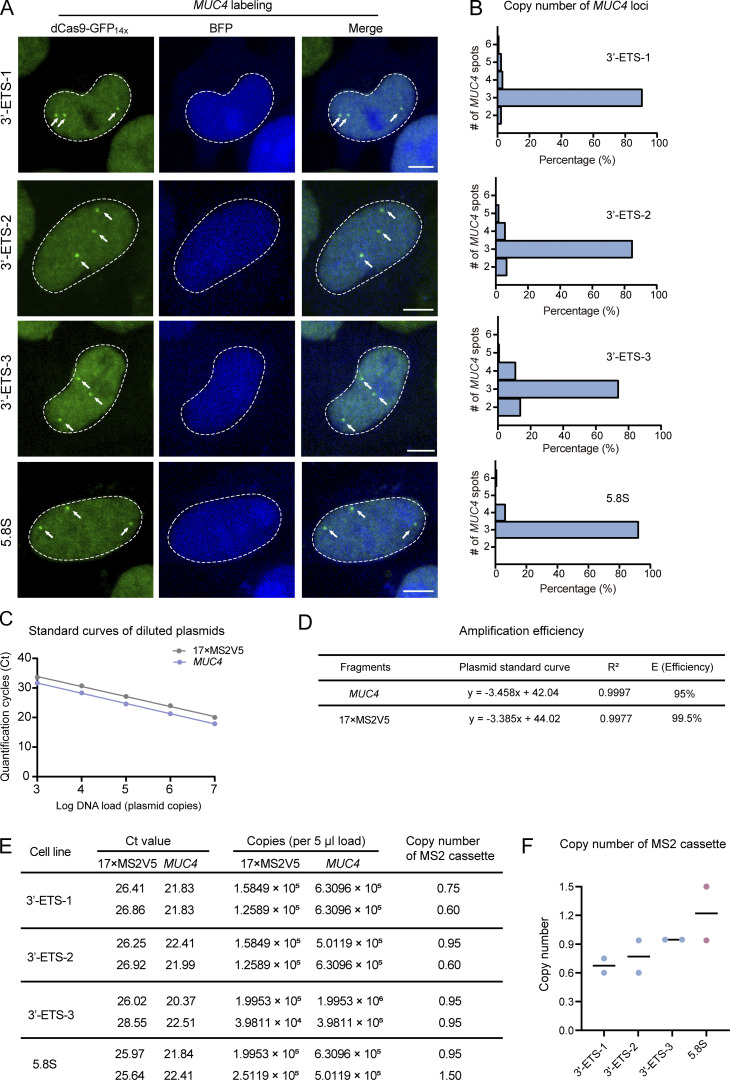
**Estimation of MS2V5**_**17X**_ **copy number in the genomic DNA of LiveArt clonal cells. (A)** Representative images to show *MUC4* labeling in four LiveArt clonal cells. Nuclear-localized BFP indicates the expression of sgRNA targeting *MUC4*. Arrows pointed to *MUC4* loci labeled by dCas9-GFP_14X_. **(B)** Histograms of *MUC4* loci number quantified by CRISPR imaging. **(C)** qPCR standard curves were generated using plasmids p*MUC4* and pMS2V5_17x_ as template. Regression curves of the 10-fold serial dilutions are presented with respect to the log of the DNA load (number of plasmid copies) added to the reaction mixture versus Ct (mean of triplicate samples). **(D)** Parameters obtained from standard curves in C were used to calculate and assess the PCR efficiency. The regression curves of the log of the plasmid load versus Ct were nearly linear (R^2^ > 0.99). The efficiency (E) of the qPCR was calculated using the equation E = 10^1/−m^ −1, where m is the slope of the line. To pass validation, the efficiency must be >90%. **(E)** qPCR results and calculated copy numbers. *n* = 2 biological replicates. **(F)** Copy number of MS2 cassette in LiveArt clonal cell lines. Black line indicates the mean value.

We then assessed how many copies of rDNA were successfully inserted with MS2 sequence in the clonal cells. The copy number of human *MUC4* gene in HeLa cells could be precisely determined by CRISPR imaging in our previous studies (Chen et al., 2013). dCas9-GFP_14X_ labeling directed by sgRNA targeting *MUC4* revealed that all clonal cells contain three copies of *MUC4* gene (Fig. S3, A, and B), which is consistent with our previous karyotype analysis (Chen et al., 2013). Therefore, we chose *MUC4* gene as the internal reference gene to calculate the relative copy number of MS2 cassettes in the target genome. Quantitative PCR (qPCR) assays suggest that only one copy of rDNA was modified by MS2 insertion in 5.8S and 3′-ETS clones (Fig. S3, C–F). However, we were not able to further characterize MS2 insertion in ITS1 clone due to its low amplification efficiency.

rDNA is first transcribed into a large pre-rRNA, which undergoes sequential cleavage to yield mature rRNAs (Henras et al., 2015). 3′-ETS and ITS1 are sequentially cleaved during this process. Therefore, MS2-tagged 3′-ETS and ITS1 rRNAs represent newly synthesized pre-rRNAs, while MS2-tagged 5.8S rRNAs consist of both pre-rRNAs and mature rRNAs. Fluorescent imaging indicated that MS2-tagged rRNAs colocalized well with nucleolar makers and rDNA labeled by dCas9-GFP_14X_ (Chen et al., 2018).

**Figure 2.**
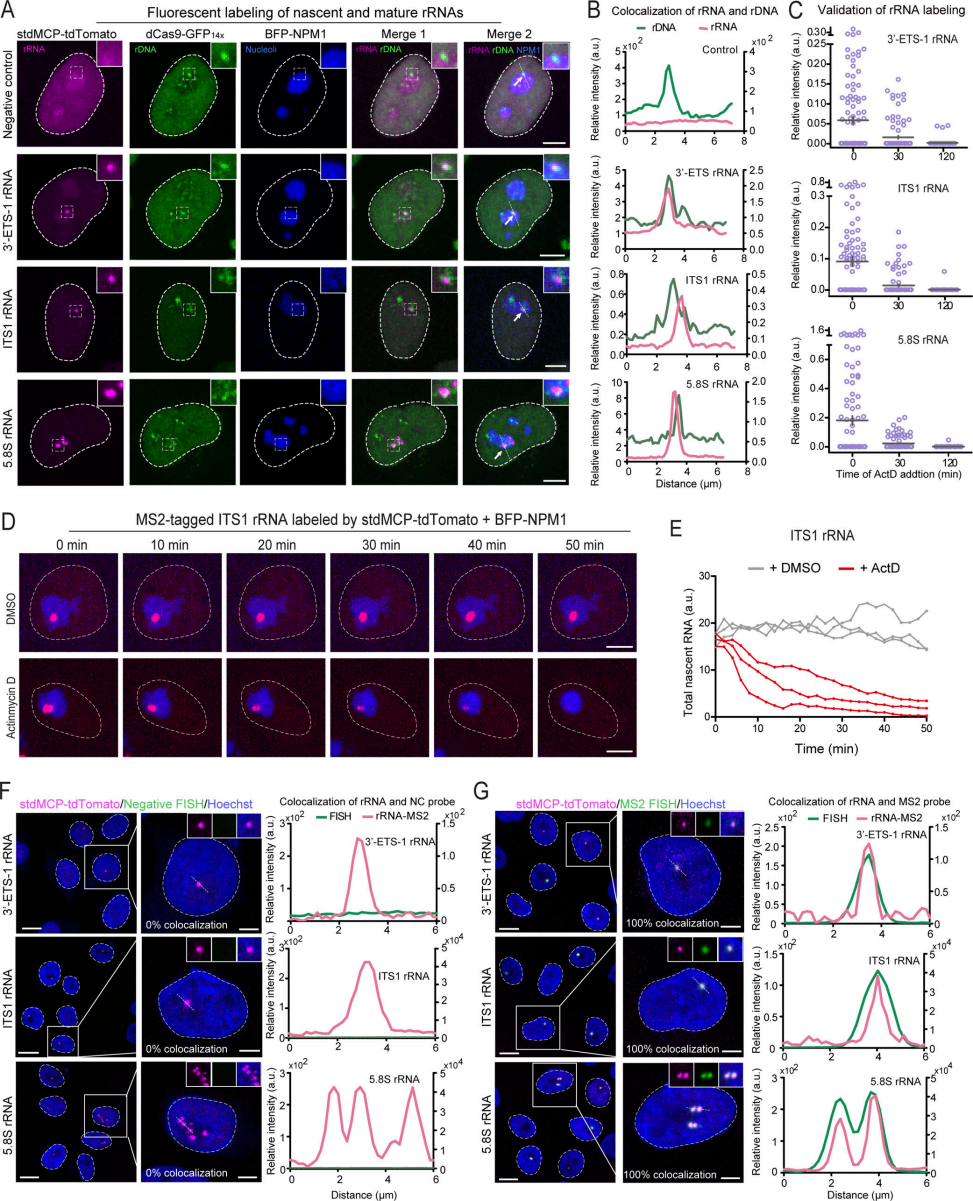
**Live-cell imaging of ribosomal RNAs via LiveArt. (A)** Representative images to show the co-localization of MS2-tagged rRNAs with rDNA and GC marker NPM1 in clonal HeLa cells. Fluorescently labeled 3′-ETS (from clone 1, named 3′-ETS-1), ITS1, and 5.8S rRNA were shown in the second, third, and fourth rows, respectively. Insert magnification: 5.2×. **(B)** Line scan of the relative fluorescence of the signal indicated by the dotted lines in A. **(C)** Quantification of MS2-tagged rRNA accumulation in the three rRNA tagging clones (3′-ETS-1, ITS1, and 5.8S) revealed by the total intensity of stdMCP-tdTomato spots in the absence or presence of ActD. Each dot represents a single cell (*n* = 100). Black line indicates mean ± SEM. **(D)** Live-cell imaging snapshots of cells showing accumulation of ITS1 rRNA indicated by stdMCP-tdTomato in the absence or presence of ActD. BFP-NPM1 was imaged to reveal the nucleoli. See Video 1 for dynamics. **(E)** Quantitative analysis of three representative cells (including one from D) showing the dynamic change of ITS1 rRNA defined by the total intensity of stdMCP-tdTomato spots in the absence or presence of ActD, respectively. **(F and G)** Representative images and line scan of fluorescent intensity showing the colocalization of stdMCP-tdTomato labeled rRNA signal (red) and RNA-FISH (green). RNA-FISH was performed using probes that do not recognize (F, negative control) or specifically recognize (G) MS2-tagged rRNAs. Colocalization ratios are indicated on the corresponding images. *n* ≥ 50 cells. All images in Fig. 2 are maximum intensity projections from *z* stacks. Scale bars, 10 µm (large-field image) and 5 µm (single-cell image).

We observed that MS2-tagged 3′-ETS or ITS1 rRNAs were enriched almost invariably as a single fluorescent spot, indicating the rDNA transcriptional site (Fig. 2, A and B). The concentration of MS2-tagged 5.8S rRNAs in either a single or multiple sites in the nucleoli may represent differential locations of 5.8S rRNAs at different stages of transcription, processing, or assembly into ribosomal units.

**Figure S4.**
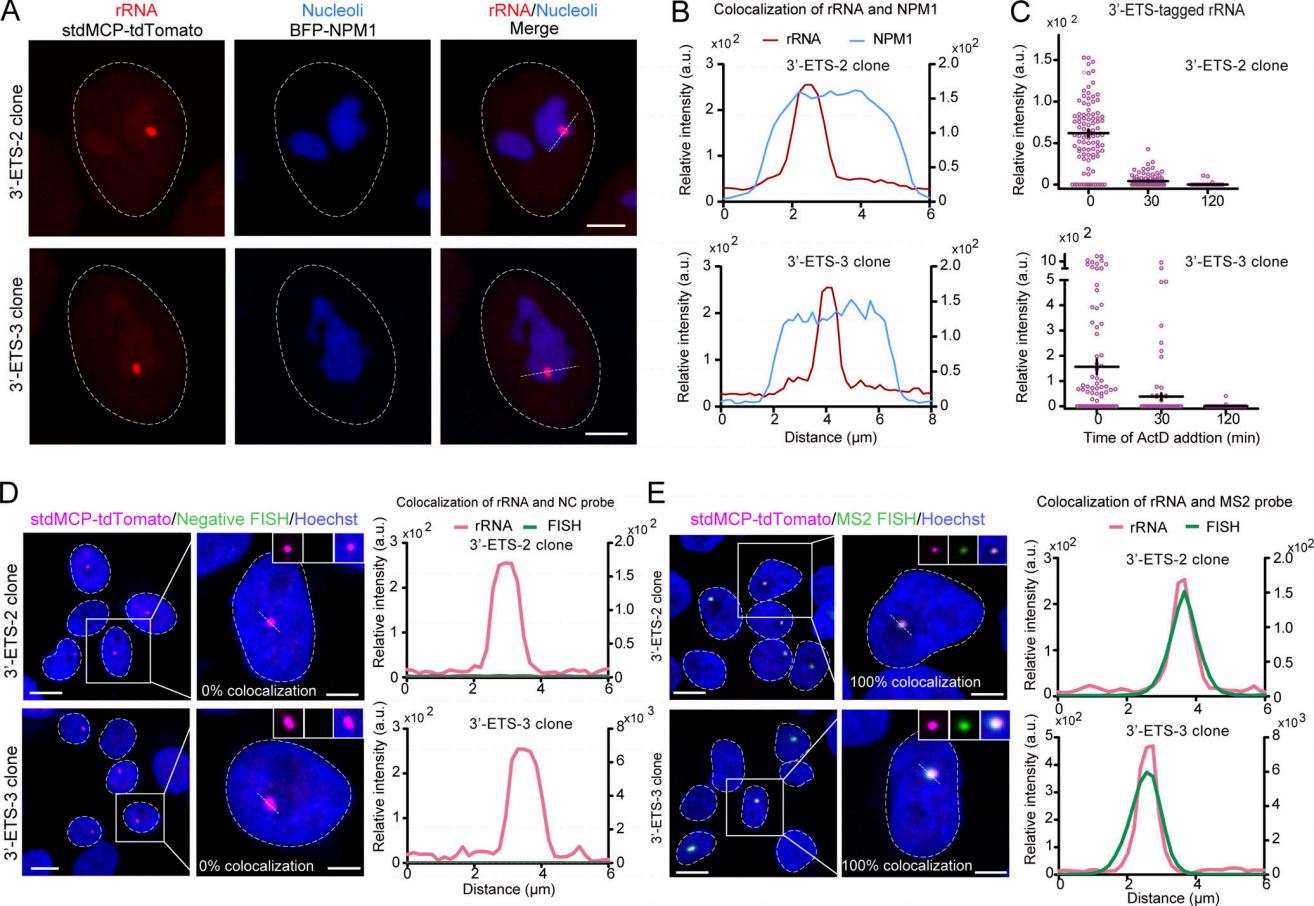
**Characterizations of additional 3′-ETS clones. (A)** Representative images to show the specific location of MS2-tagged rRNAs labeled by stdMCP-tdTomato in the nucleoli labeled by BFP-NPM1. Scale bar, 5 µm. **(B)** Line scan of the relative fluorescence of the signal indicated by the dotted lines in A. **(C)** Quantification of MS2-tagged rRNA accumulation in 3′-ETS clones (3′-ETS-2, 3′-ETS-3) by quantifying the total intensity of stdMCP-tdTomato spots in the absence or presence of ActD. Each dot represents a single cell (*n* ≥ 100). Data are displayed as mean ± SEM. **(D and E)** Left: Representative images to show the co-localization of stdMCP-tdTomato (red) with RNA-FISH (green) using FISH probes that could not recognize MS2- tagged rRNAs (D) or could specifically bind to MS2V5_17X_ in MS2-tagged rRNAs (E) in 3′-ETS clones. Colocalization ratios are indicated on the corresponding images. *n* ≥ 53 cells. Right: Line scan of the relative fluorescence of the signal indicated by the dotted lines in the images. All images are maximum-intensity projections from *z* stacks. Nuclei are outlined with white circles. Scale bars, 10 µm (large-field image) and 5 µm (single-cell image).

It is well established that a low concentration of Actinomycin D (ActD) selectively inhibits rRNA synthesis (Iapalucci-Espinoza and Franze-Fernandez, 1979). We therefore used this drug to further validate the specificity of our rRNA imaging system. Quantitative analysis and real-time tracking indicated that the accumulation of MS2-tagged rRNAs was indeed gradually reduced following ActD treatment (Fig. 2, C–E, Fig. S4, A–C, and Video 1). Notably, we observed that 100% of stdMCP-tdTomato labeled rRNA foci could be specifically stained by RNA-FISH probes that recognize MS2 sequences, indicating that stdMCP-tdTomato signal is highly specific for detecting MS2-tagged rRNAs (Fig. 2, F and G, and Fig. S4, D and E). Together, our results suggest that 3′-ETS clones, which likely generated fluorescent nascent rRNAs from single rDNA locus, can be used to analyze transcriptional bursting of rDNA. While 5.8S and ITS1 clones could be applied to quantitatively assess the change of Pol I transcriptional activity. Taken together, these results demonstrated a quantitative rRNA tagging system termed LiveArt (live imaging-based analysis of rDNA transcription).

**Video 1.** **Validation of ITS1 rRNA labeling in clonal cells.** ActD was applied to inhibit Pol I transcription in the clonal cells harboring MS2-tagged ITS1 rRNA. stdMCP-tdTomato indicates newly produced ITS1 rRNAs. 50-min video is shown. Images were acquired with 6 *z*-planes spaced by 1.0 μm every 2 min. Scale bar, 2 μm. Playback, 10 frames per s (fps).

### Functional evaluation of LiveArt clonal cells

**Figure S5.**
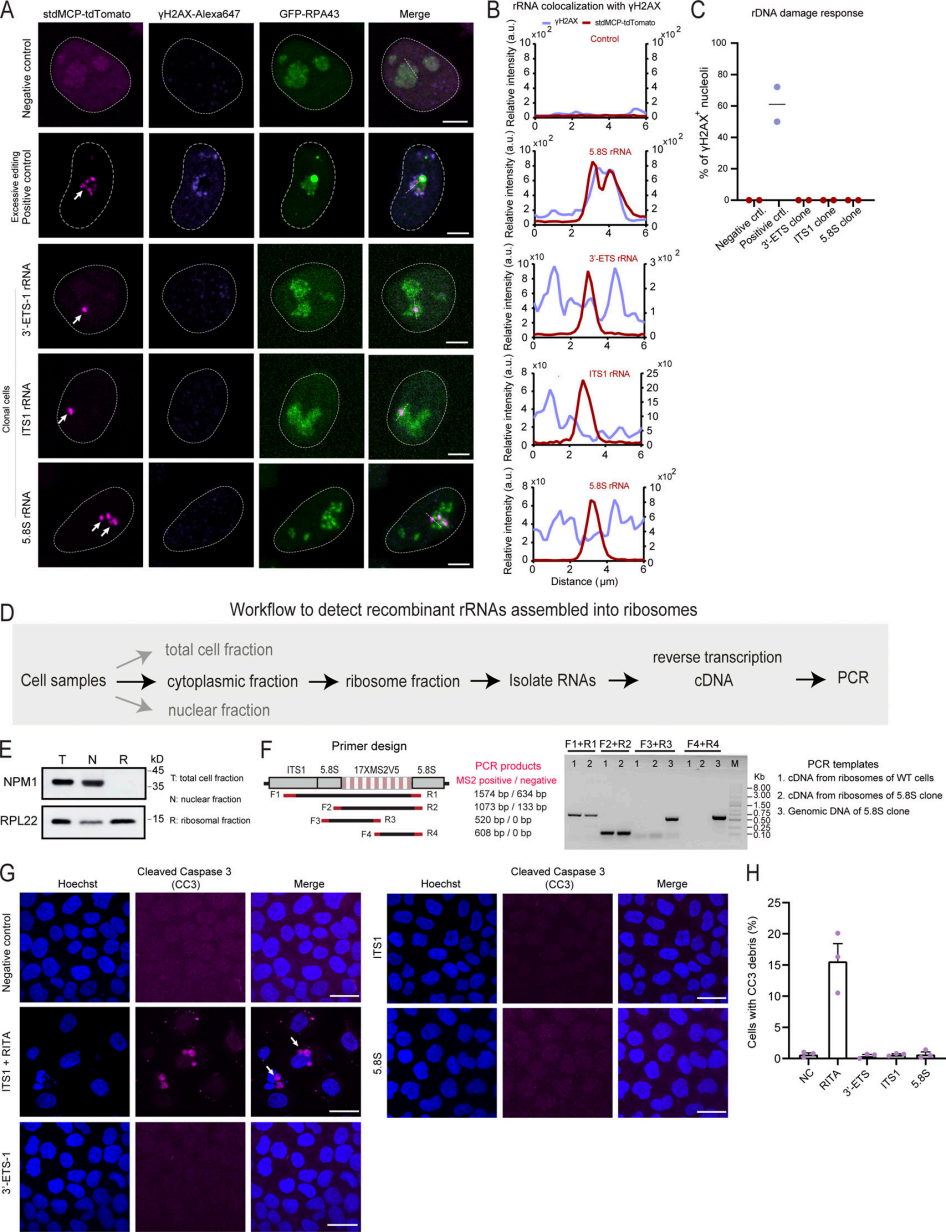
**Detection of MS2-tagged rRNAs in ribosomes and DNA breaks or apoptosis in LiveArt clonal cells. (A)** Representative images to show the colocalization of γH2AX immunofluorescence with MS2-tagged rRNAs indicated by stdMCP-tdTomato in LiveArt clonal cells (3′-ETS-1, ITS1, and 5.8S). 5.8S rRNA tagging by transient transfection (excessive rDNA editing) was shown as a control (second row), in which γH2AX are present in the nucleolus. All images are maximum intensity projections from z stacks. Scale bars, 5 µm. Arrows point to visible MS2-tagged rRNAs. **(B)** Line scan of the relative fluorescence of the signal indicated by the dotted lines in A. **(C)** Percentage of nucleoli harboring enriched γH2AX signal under different labeling conditions in A. *n* = 2 biological replicates. Black line indicates the mean value. **(D)** Schematic illustration of the workflow to detect MS2-tagged rRNAs in ribosomes using PCR. **(E)** Western blot to detect nucleolar protein NPM1 or ribosomal protein RPL22 for demonstrating the success to isolate ribosomal fractions with high purity. **(F)** Primer designs and the corresponding PCR products to show whether MS2 cassette was present in the template DNA. **(G)** Representative images to show the colocalization of CC3 immunofluorescence with cell nucleus indicated by Hoechst 33342 in wild-type cells (stdMCP-tdTomato stable cell line without rRNA tagging) and LiveArt clonal cells (3′-ETS-1, ITS1, and 5.8S). ITS1 clone treated with RITA to induce apoptosis was shown as a positive control (second row), in which CC3 signal was condensed. All images are maximum-intensity projections from *z* stacks. Scale bars, 10 µm. Arrows point to condensed CC3 signal. **(H)** The percentage of CC3 positive rate under different conditions in G. *n* = 3 biological replicates. Data are shown as mean ± SEM. Source data are available for this figure: SourceData FS5.

**Figure 3.**
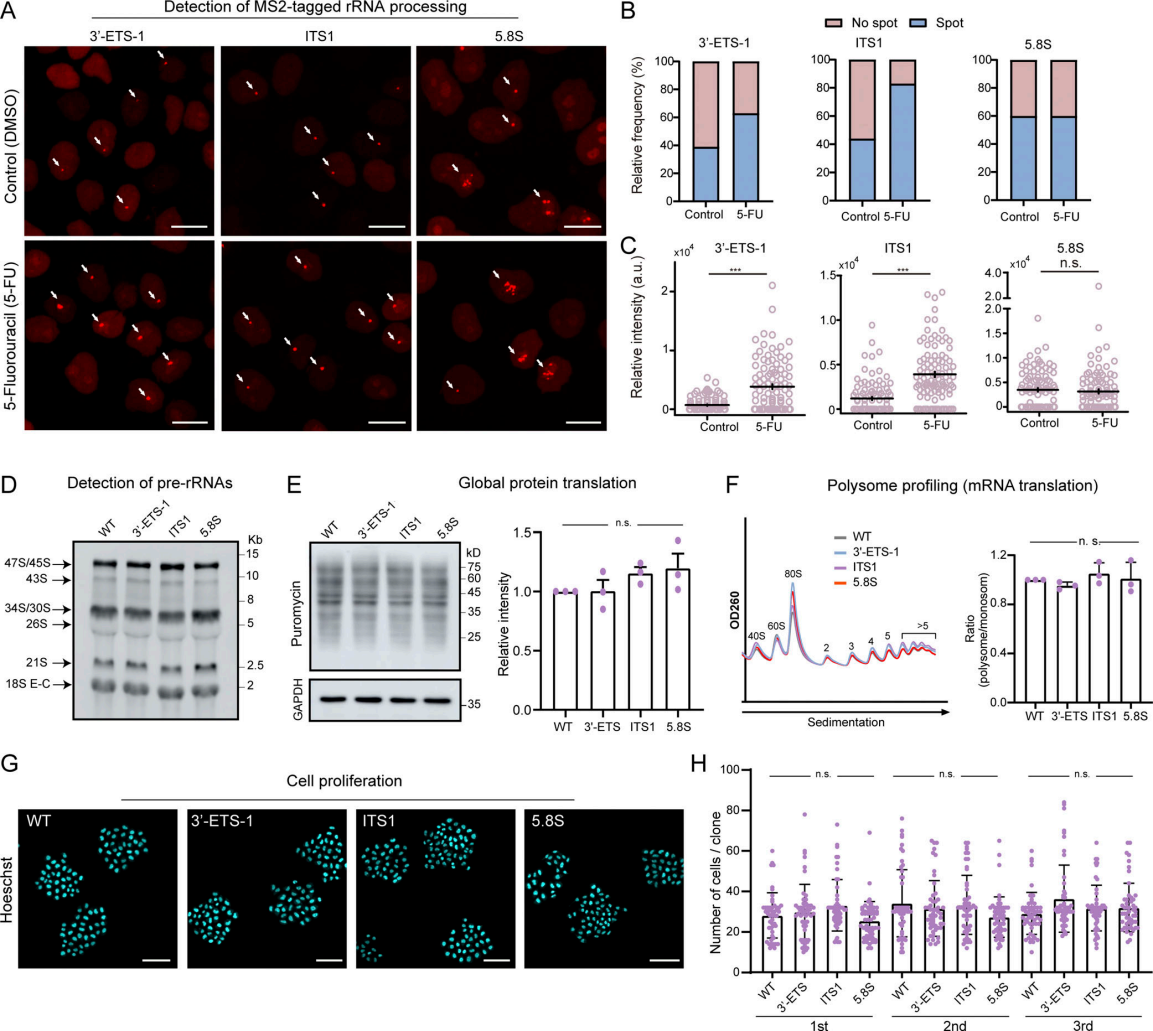
**Functional evaluation of LiveArt clonal cell lines. (A)** Representative images illustrating the changes of stdMCP-tdTomato labeled rRNAs when treated with 5-FU in LiveArt clonal cells (3′-ETS-1, ITS1, and 5.8S). All images are maximum-intensity projections from *z* stacks. Scale bars, 10 µm. Arrows point to visible MS2-tagged rRNAs. **(B)** Bar graph showing the percentage of cells with visible rRNA indicated by stdMCP-tdTomato in the absence or presence of 5-FU (*n* ≥ 124). **(C)** Total intensity of visible stdMCP-tdTomato spots in each cell was measured under different conditions to generate the plot. Each dot represents a single cell (*n* = 100). Data are displayed as mean ± SEM. Two-tailed paired *t* test, ***P ≤ 0.001. **(D)** Analysis of pre-rRNA processing by Northern blotting. stdMCP-tdTomato stable cell line without rRNA tagging was used as the wild-type control (WT, lane 1), which are the parental cells of the three LiveArt clonal cell lines (lanes 2–4). Probe hybridized to ITS1 region was used for detection. **(E)** Left: Puromycin incorporation into control and LiveArt cells detected by anti-puromycin blot. GAPDH immunoblot is shown as a loading control (bottom). Right: Graphs show the quantifications of puromycin incorporation, indicating mean ± SEM (*n* = 3 replicates). **(F)** Left: Representative examples of polysome profiles from WT and LiveArt clonal cells. Right: Polysome abundance analysis of profiles performed in left. The ratios of polysome to monosome are presented as mean ± SEM (*n* = 3 replicates). **(G)** Representative images illustrating the formation of clonal cells after growing from a single cell for 5 d. Cell nuclei were stained by Hoechst 33342. All images are from single focal plane. Scale bars, 100 µm. **(H)** Quantification of the number of cells per clone in different cell lines in E (*n* = 3 replicates). Each dot represents a single clone (*n* ≥ 50). Data are displayed as mean ± SEM. n.s., not significant. One-way ANOVA with Tukey’s post hoc was used to test differences between groups. Source data are available for this figure: SourceData F3.

Next, we performed a series of assays to evaluate whether inserting MS2 repeats inside rDNA impact ribosome biogenesis and cellular function in representative LiveArt clonal cells (ITS1, 5.8S, and 3′-ETS-1 clones). To examine whether CRISPR editing induced excessive DNA breaks in rDNA clusters, we applied immuno-staining to detect γ-H2AX (phosphorylated H2AX), a well-established DNA damage marker (Rogakou et al., 1998). We did not observe a significant accumulation of γ-H2AX signal in the nucleoli (Fig. S5, A–C). The chemotherapeutic agent 5-Fluorouracil (5-FU) has been shown to block normal pre-rRNA processing (Ghoshal and Jacob, 1994). We observed that 5-FU treatment resulted in increased accumulation of 3′-ETS and ITS1 rRNAs (brighter rRNA foci and a greater proportion of cells with visible rRNAs), but not an increase in 5.8S (Fig. 3, A–C). Thus, our data suggest that MS2-tagged 3′-ETS and ITS1 can indeed be processed. Moreover, Northern blot analysis suggested that rRNA processing was not obviously affected (Fig. 3 D).

We next employed the puromycin incorporation assay to determine the global protein synthesis rates and found no significant differences between wild-type and LiveArt cells (Fig. 3 E). We also examined bulk translation status by utilizing polysome profiling, which assess translation by comparing the relative distribution of monosomes and polysomes (Chasse et al., 2017). Polysome profiling revealed no differences in the translation efficiencies between wild-type cells and rRNA tagging cells (Fig. 3 F). We then evaluated whether MS2-tagged 5.8S rRNA is incorporated into ribosomes. Toward this goal, the ribosome fraction was specifically isolated from 5.8S clonal cells. The total RNA associated with ribosomes were then purified and reversely transcribed into DNA for use as a PCR template. Using PCR assays, we could not detect the presence of MS2-tagged 5.8S rRNA in the ribosome fraction, which is consistent with our observation that MS2-tagged 5.8S signal did not appear in the cytoplasm (Fig. S5, D–F). There are two possible explanations for these results: (1) The proportion of MS2-tagged 5.8S rRNA in the total rRNA was very low, which may be below the detection sensitivity of PCR; (2) after being assembled into ribosomes, MS2-tagged 5.8S rRNA may not be able to bind to stdMCP-tdTomato. Regardless of the conclusions, whether MS2-tagged 5.8 rRNA is properly incorporated into ribosomes does not affect its role as a reporter of Pol I activity.

Then, we sought to assess cell growth and proliferation activity of the three rRNA tagging clones. Cleaved caspase-3 (CC3) is considered a reliable marker for detecting apoptotic cells (Crowley and Waterhouse, 2016). We found that CC3 staining could label apoptotic cells induced by the small drug RITA (reactivation of p53 and induction of tumor cell apoptosis) treatment but did not detect increased apoptotic proteins in LiveArt cells (Fig. S5, G and H). Furthermore, we performed colony formation assay to evaluate the proliferation activity of LiveArt cells. After 5 d of cell culture, we counted the number of cells in each single cell clone. The quantitative results demonstrated that LiveArt cells still retain similar proliferation activity as wild-type cells (Fig. 3, G and H). Taken together, our data reveal that our LiveArt clonal cells did not significantly impair protein translation and cell proliferation.

### Monitoring the effect of rRNA synthesis on nucleolar reassembly during mitosis through LiveArt

**Figure 4.**
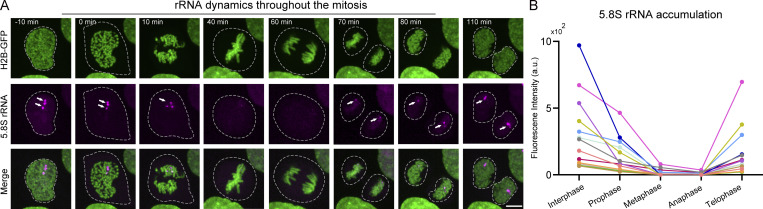
**Pol I transcription switches off and on during mitosis. (A)** Live-cell imaging snapshots to show dynamic changes of 5.8S rRNA labeled by stdMCP-tdTomato and H2B-GFP during mitosis. “0 min” indicates the time of nuclear membrane rupture. See Video 2 for dynamics. The white circle highlights the area where the stdMCP-tdTomato is located, which represents the outline of the nucleus (before nuclear membrane rupture) or the cell (after nuclear membrane rupture). Arrows point to visible MS2-tagged rRNAs. **(B)** Broken line diagram illustrating the dynamic change of rRNA synthesis during the cell cycle. Each line represents a single cell (*n* = 13).

Nucleolar architecture undergoes extensive changes throughout the mitosis, during which rDNA transcription is repressed and reactivated (Nemeth and Grummt, 2018; Weisenberger and Scheer, 1995; Zharskaia and Zatsepina, 2007). Using FISH or BrUTP incorporation to detect pre-rRNAs in fixed cells, it has been estimated that rRNA synthesis generally pauses in metaphase and restarts at late anaphase or early telophase (Fomproix et al., 1998; Hernandez-Verdun et al., 2002; Leung et al., 2004; Morcillo et al., 1976; Roussel et al., 1996). However, there are no appropriate methods for analyzing spatiotemporal dynamics of rRNA synthesis throughout mitosis by tracing single cells. We performed time-lapse imaging of 5.8S clone co-expressing GFP-RPA43, Halo-FBL, and BFP-NPM1. Real-time quantitative imaging revealed that rDNA transcription was gradually shut down as the cell progressed from interphase to metaphase and reactivated as the cell was entering telophase from anaphase (Fig. 4 and Video 2). Thus, the fate of rRNAs during mitosis can be quantitatively analyzed in space and time through LiveArt.

**Video 2.** **Dynamic changes of rRNA synthesis throughout mitosis.** Simultaneous live-cell imaging of 5.8S rRNA and H2B-GFP throughout mitosis. H2B-GFP (green) was imaged to reveal the mitotic process. stdMCP-tdTomato (red) revealed the dynamic changes of rRNA synthesis in real time. 150-min video is shown. Images were acquired with 11 *z*-planes spaced by 0.6 μm every 5 min. Scale bar, 5 μm. Playback, 10 fps.

**Figure 5.**
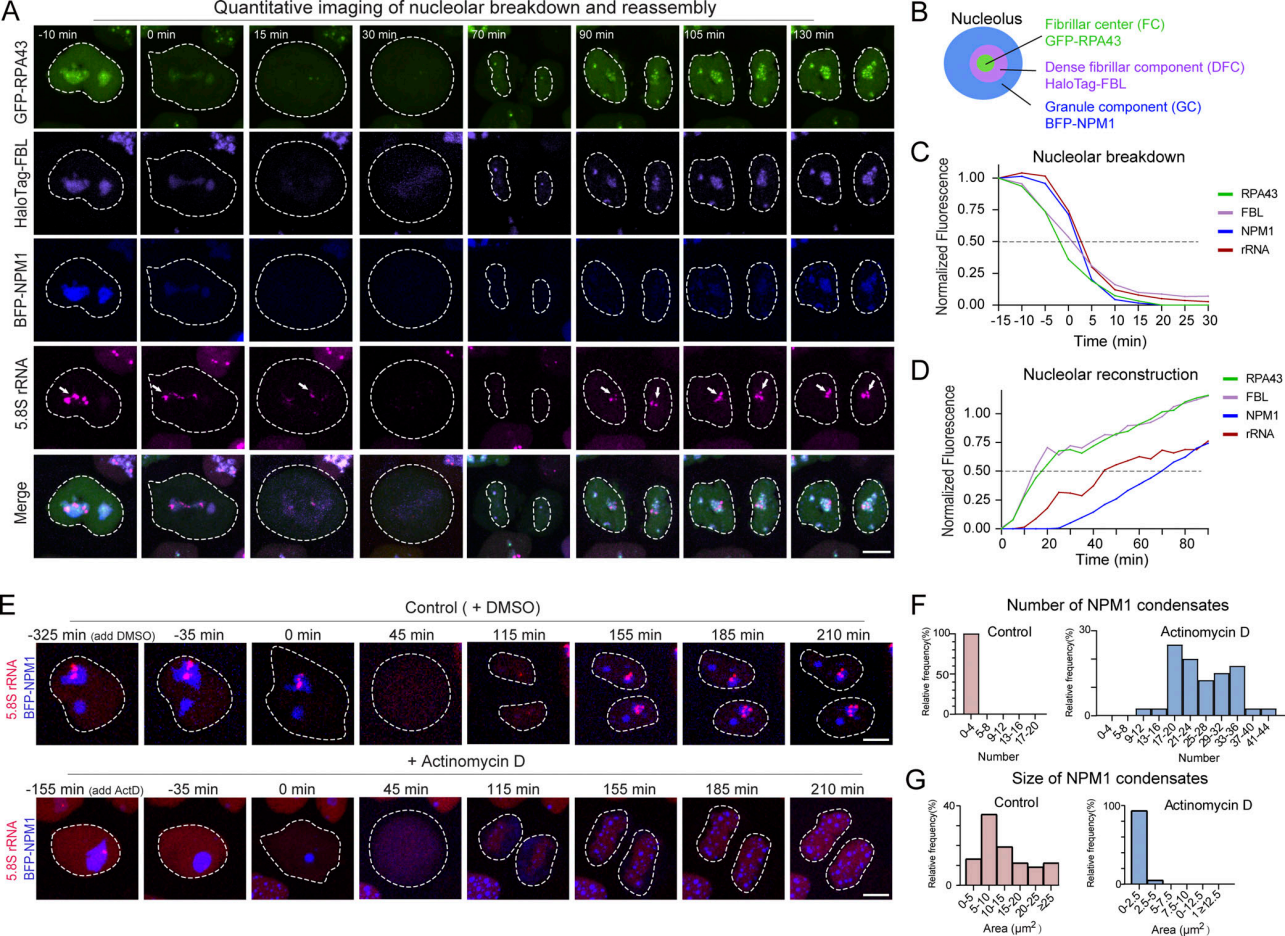
**Pol I reactivation is crucial for nucleolar reconstruction during mitosis. (A)** Snapshots of live-cell imaging of 5.8S rRNA and nucleolar markers during mitosis. The time shown on the top of each panel indicates the time for the cell progressing through mitosis, and “0 min” indicates the time of nuclear membrane rupture. Arrows point to visible MS2-tagged rRNAs. **(B)** Schematic of the nucleolar architecture and the markers used to label nucleolar subcompartments in A. **(C)** The mean fluorescence intensities of nucleolar components RPA43, FBL, NPM1, and 5.8S rRNA were averaged, normalized, and plotted against time, where “0 min” is the time of nuclear membrane rupture (*n* = 15 cells). **(D)** The mean fluorescence intensities of nucleolar components RPA43, FBL, NPM1, and 5.8S rRNA within the reforming nuclei were averaged, normalized, and plotted against time, where time equals “0 min” when the chromosomes start to migrate toward respective poles (*n* = 15 cells). **(E)** Snapshots of live-cell imaging of MS2-tagged 5.8S rRNA labeled by stdMCP-tdTomato and BFP-NPM1 during mitosis in the absence or presence of ActD. The first image shown in each row is the first image taken intermediately after adding DMSO or ActD into the medium. “0 min” is the time of nuclear membrane rupture. See Videos 3 and 4 for dynamics. **(F)** Histograms illustrating the number of BFP-NPM1 condensates formed in the daughter cells upon mitosis exit in the absence or presence of ActD (*n* = 40). **(G)** Histograms demonstrating the size of the BFP-NPM1 condensates formed in the daughter cells upon mitosis exit in the absence or presence of ActD (*n* ≥ 98). All images in this figure are maximum-intensity projections from *z* stacks. The white circle highlights the area where the stdMCP-tdTomato is located, which represents the outline of the nucleus (before nuclear membrane rupture) or the cell (after nuclear membrane rupture). Scale bars, 5 µm.

We then monitored rRNA dynamics during nucleolar breakdown and reassembly in HeLa cells. We quantitated the temporal pathway of nucleolar breakdown and reassembly by measuring the levels of fluorescent protein in defined nucleolar regions. Loss of RPA43 and FBL in the nucleolus preceded that of rRNA and NPM1. By comparing the timing of 50% signal loss in nucleoli, we found that significant loss of RPA43 occurred ∼ 2.5 min before FBL and ∼ 5 min before NPM1 and rRNA. Notably, the dramatic loss of NPM1 and rRNA from nucleoli occurred nearly simultaneously (Fig. 5, A–C). Next, we analyzed the rate of fluorescence increase over time within reforming nucleoli. RPA43 and FBL assembled into nucleoli nearly simultaneously. Moreover, the assembly of FC and DFC occurred earlier than GC, which is consistent with previous data (Leung et al., 2004). Our quantitative measurements indicated rDNA reactivation occurs ∼10 min after the initial enrichment of RPA43 and FBL but ∼15 min before GC formation, illustrating a stepwise reformation of nucleoli (Fig. 5, A and D). These 4D imaging data provide the first dynamic view of rRNA synthesis associated with nucleolar breakdown and reformation in single cells.

To further test the role of rRNA synthesis in nucleolar reassembly, cells were treated with ActD to block rDNA reactivation. Quantitative analysis showed that a lack of rRNA caused the presence of more but smaller BFP-NPM1 puncta relative to untreated cells (area size of puncta: 1.24 vs. 12.92 µm^2^; number of puncta: 25.5 vs. 2.5). Nucleolar structures could not be normally formed in these cells at the end of mitosis (Fig. 5, E–G; and Videos 3 and 4). Our direct observation supports the idea that interactions between NPM1 and rRNA are involved in integrating NPM1 within the GC matrix (Feric et al., 2016; Mitrea et al., 2016; Mitrea et al., 2018).

**Video 3.** **Dynamics of nucleolar reassembly at the end of mitosis in the absence of ActD.** Simultaneous live-cell imaging of MS2-tagged 5.8S rRNA labeled by stdMCP-tdTomato and BFP-NPM1 throughout mitosis in the absence of ActD. 6-h video is shown. Images were acquired with 11 *z*-planes spaced by 0.6 μm every 5 min. Scale bar, 5 μm. Playback, 10 fps.

**Video 4.** **Dynamics of nucleolar reassembly at the end of mitosis in the presence of ActD.** Simultaneous live-cell imaging of MS2-tagged 5.8S rRNA labeled by stdMCP-tdTomato and BFP-NPM1 throughout mitosis in the presence of ActD. 6-h video is shown. Images were acquired with 11 *z*-planes spaced by 0.6 μm every 5 min. Scale bar, 5 μm. Playback, 10 fps.

### Dynamic link between rDNA transcription and nucleolar integrity in interphase

**Figure 6.**
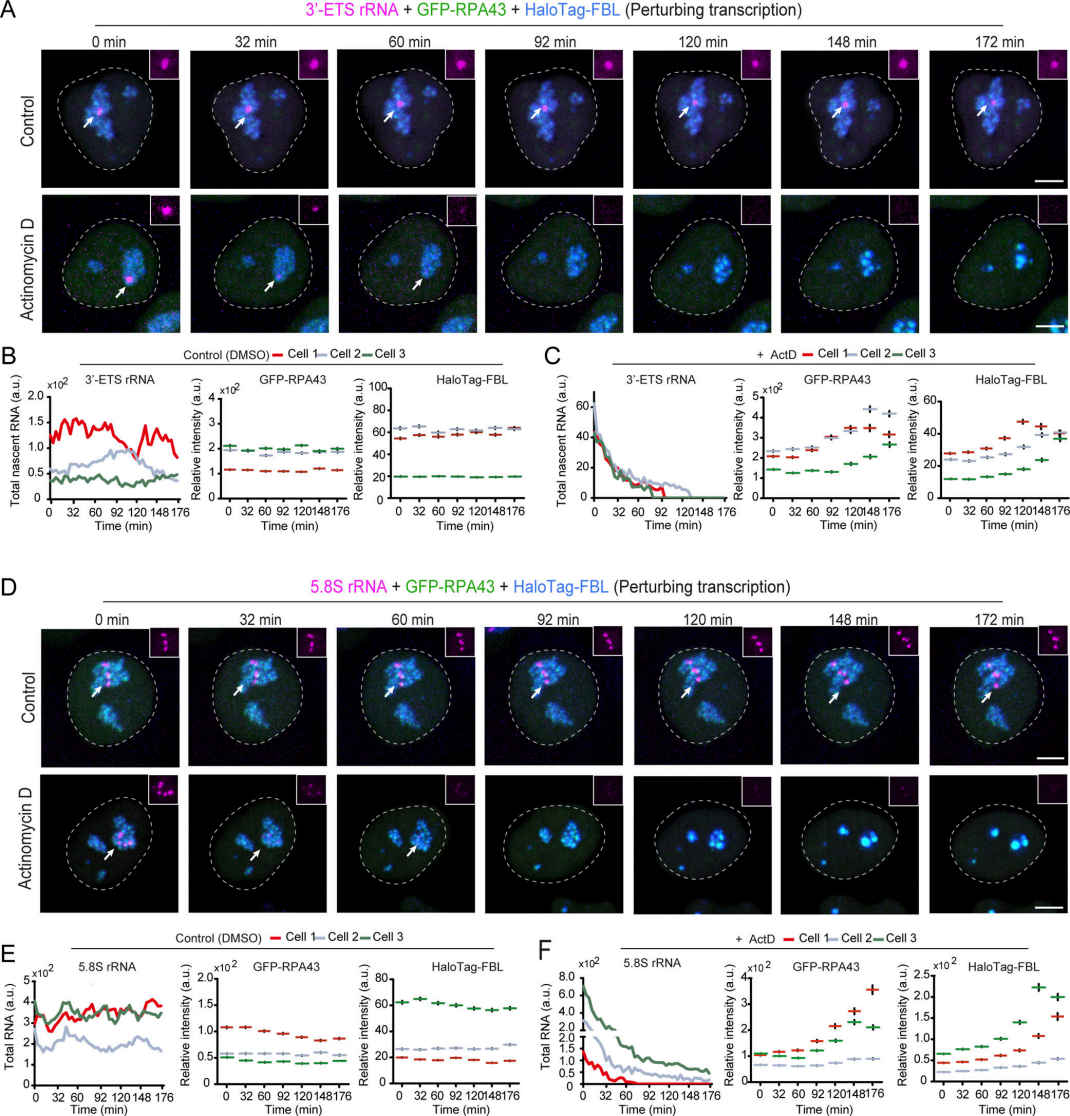
**LiveArt monitors the dynamic process of nucleolar segregation induced by Pol I transcriptional shut-down in interphase. (A)** Snapshots of live-cell imaging of 3′-ETS-1 rRNA and nucleolar markers changes in the absence or presence of ActD. FC is labeled by GFP-RPA43 (green) and DFC is indicated by HaloTag-FBL (blue). Arrows point to the nucleolus that harbors active rRNA synthesis. The accumulation of MS2-tagged rRNAs indicated by stdMCP-tdTomato was highlighted with a white box. Insert magnification: 1.2×. See Videos 5 and 6 for dynamics. **(B and C)** Quantitative analysis of three representative cells (including one from A) showing dynamic changes in 3′-ETS-1 rRNA defined by the total intensity of stdMCP-tdTomato spots, as well as GFP-RPA43 or HaloTag-FBL by counting the mean fluorescence intensity of the regions (*n* ≥ 179) where they were enriched in the absence (B) or presence (C) of ActD. **(D)** Snapshots of live-cell imaging showing the dynamic changes of 5.8S rRNA and nucleolar markers in the absence or presence of ActD. Insert magnification: 0.54×. Arrows point to the nucleolus that harbors active rRNA synthesis. **(E and F)** Quantifications of three representative cells (including one from D) showing the dynamic change of 5.8S, GFP-RPA43, and HaloTag-FBL (analyzed in the same way as B and C, *n* ≥ 119) in the absence (E) or presence (F) of ActD. All images in Fig. 6 are maximum-intensity projections from *z* stacks. Nuclei are outlined with white circles. The data of RPA43 and FBL at each time point are represented as mean ± SEM. Scale bars, 5 µm.

It is reported that a block of rDNA synthesis leads to the reorganization of interphase nucleoli (Floutsakou et al., 2013; Frottin et al., 2019). However, this dynamic process has not been monitored with appropriate rRNA reporters in living cells. Therefore, we combined LiveArt and FC/DFC markers to capture detailed information about the dynamics of nucleolar reorganization in the 3′-ETS-1 clone. We measured the mean intensity of GFP-RPA43 and HaloTag-FBL signals enriched in the nucleolus, as well as the total amount of MS2-tagged 3′-ETS produced in each nucleolus. Quantitative imaging indicates that in the control cells MS2-tagged rDNA was normally transcribed and the mean intensity of FCs/DFCs remained stable. Upon ActD treatment, the accumulation of 3′-ETS rRNA decreased rapidly and was reduced by ∼90% after 60 min. Intriguingly, FCs and DFCs were not significantly altered at this time point (Fig. 6, A–C; and Videos 5 and 6). In contrast, starting ∼90 min after ActD treatment, the nucleoli underwent obvious shrinkage and dramatic remodeling, often referred to as nucleolar segregation (Nemeth and Grummt, 2018; van Sluis and McStay, 2017). Consistently, 5.8S clone imaging confirmed similar dynamic changes (Fig. 6, D–F). Overall, our observations reveal that spatial segregation of FCs/DFCs occurs after transcriptional shutdown and the disappearance of most mature rRNAs. These results again support the role of rRNAs in assembling and maintaining nucleolar integrity.

**Video 5.** **Dynamics of nucleolar structure in interphase.** Simultaneous live-cell imaging of 3′-ETS rRNA and nucleolar components in the absence of ActD. MS2-tagged 3′-ETS-1 rRNA was indicated by stdMCP-tdTomato (red). GFP-RPA43 (green) was imaged to reveal the FC, whereas Halotag-FBL (blue) was imaged to reveal the DFC. Cells were treated with DMSO as a negative control. 3-h video is shown. Images were acquired with 6 *z*-planes spaced by 1 μm every 4 min. Scale bar: 2 μm. Playback, 10 fps.

**Video 6.** **Dynamics of nucleolar structure in interphase upon ActD treatment.** Simultaneous live-cell imaging of 3′-ETS-1 rRNA and nucleolar components in the presences of ActD. MS2-tagged 3′-ETS rRNA was indicated by stdMCP-tdTomato (red). GFP-RPA43 (green) was imaged to reveal the FC, whereas Halotag-FBL (blue) was imaged to reveal the DFC. 3-h video is shown. Images were acquired with 6 *z*-planes spaced by 1 μm every 4 min. Scale bar, 2 μm. Playback, 10 fps.

### Bursting kinetics of Pol I transcription in interphase

**Figure 7.**
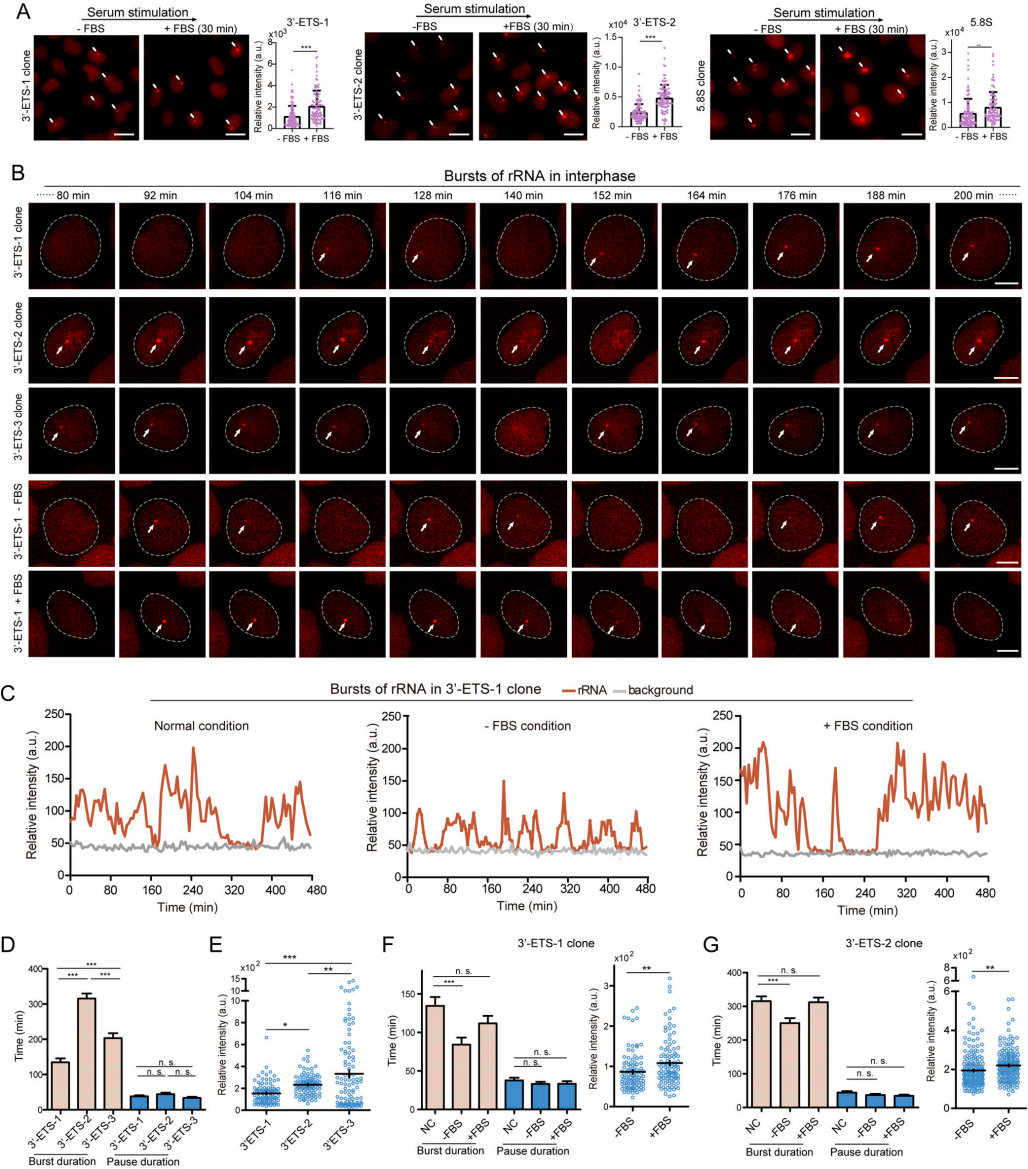
**Bursting kinetics of rDNA transcription under various conditions. (A)** Representative images illustrating the changes of MS2-tagged rRNAs (3′-ETS or 5.8S) when cells were stimulated with FBS for 30 min after serum starvation for 24 h. All images are maximum-intensity projections from *z* stacks. Scale bars, 10 μm. Total intensity of stdMCP-tdTomato spots in each cell was quantified and plotted under different conditions (*n* ≥ 100). Data are shown as mean ± SEM. Two-tailed paired *t* test, **P ≤ 0.01, ***P ≤ 0.001. Arrows point to visible MS2-tagged rRNAs. **(B)** LiveArt imaging snapshots showing transcriptional bursts of rDNA in three 3′-ETS clones under different conditions. stdMCP-tdTomato signal was imaged for 8 h (4-min interval), but only snapshots between 80 and 200 min are shown. See Video 7 for dynamics. Arrows point to visible MS2-tagged rRNAs. **(C)** Representative traces (red) illustrating the real-time synthesis of MS2-tagged rRNAs in single cells by measuring the maximum intensity of stdMCP-tdTomato spots at all time points. Gray traces denote the background signal. **(D)** Histograms of burst and pause durations demonstrating the transcriptional bursting of rDNA in three 3′-ETS clones at normal conditions. *n* ≥ 100. **(E)** Quantification of burst amplitude defined by the maximum total intensity of stdMCP-tdTomato spots in a burst. *n* ≥ 100. **(F and G)** Quantification of burst duration, pause duration, and burst amplitude by imaging 3′-ETS clone 1 and 2, respectively. *n* ≥ 100. The negative control (NC) results in F and G are the same dataset as that of 3′-ETS-1 in D. Data are all displayed as mean ± SEM. One-way ANOVA with Tukey’s post hoc was used to test differences between groups. *P ≤ 0.05, **P ≤ 0.01, ***P ≤ 0.001, n.s., not significant.

We first addressed whether LiveArt could report up-regulation of rDNA transcription. To do this, LiveArt clonal cells were stimulated with serum after serum starvation. Consistent with previous studies (de Capoa et al., 1985; Hannan et al., 2003; Xu et al., 2021), we found that serum stimulation rapidly elevated rRNA synthesis within 30 min, revealed by fluorescently labeled rRNAs (3′-ETS or 5.8S; Fig. 7 A). Next, we sought to probe how rDNA transcription levels are modulated in response to serum starvation and stimulation by measuring transcriptional bursting kinetics of MS2-tagged rDNA loci.

Quantitative RNA imaging methods illustrate that transcriptional bursting is a general property of gene expression driven by RNA polymerase II in all kinds of organisms (Lim, 2018; Suter et al., 2011). Therefore, we asked whether LiveArt could report the transcriptional kinetics of Pol I in interphase, which has not been observed in living cells before. The primary transcript of rDNA (47S pre-rRNA) is initially cleaved at both ends, including 3′-ETS (Mullineux and Lafontaine, 2012). Thus, 3′-ETS tagging can be applied as a sensitive reporter to detect 47S pre-rRNA. We performed real-time imaging to record the production of MS2-tagged 3′-ETS (hereafter referred to as 3′-ETS) in the three 3′-ETS clones we have isolated. Images were taken for 8 h at intervals of 4 min. Most stdMCP-tdTomato spots oscillated between well above (“on” state) or indistinguishable (“off” state) from the background signal. Quantitative analysis revealed that the transcription of all three tagged rDNA loci occurs in discontinuous bursts (Fig. 7, B and C; and Video 7). We then analyzed burst and pause durations to define ON- and OFF-times of rDNA transcription. The mean burst duration (ON-time) varied dramatically between the three different rDNA loci (∼134.4 min, ∼315.6 min, ∼203.1 min), whereas the mean pause duration (OFF-time) was not significantly different (∼37.7 min, ∼44.0 min, ∼33.4 min; Fig. 7 D). These observations demonstrate that active rDNA is transcribed in long bursts (ON-time) with periods of inactivity (OFF-time) between them. Additionally, burst amplitude analysis suggests that the three tagged rDNA loci produce nascent pre-rRNAs at different rates (Fig. 7 E).

**Video 7.** **Transcriptional bursting of MS2-tagged 3′-ETS rRNA in interphase.** Live-cell imaging of transcriptional bursting of MS2-tagged 3′-ETS-1 rRNA indicated by stdMCP-tdTomato in clonal cells. 8-h video is shown. Images were acquired with 6 *z*-planes spaced by 1 μm every 4 min. Scale bar, 5 μm. Playback, 10 fps.

To further assess how rDNA transcription responds to serum starvation and stimulation, we analyzed transcription bursting features of rDNA loci in 3′-ETS-1 and 3′-ETS-2 clones, respectively (Fig. 7, B and C). We found that the average ON-time of rDNA transcription was significantly reduced when the cells were starved by depletion of FBS for 24–32 h. However, when starved cells were stimulated with serum, the shortened burst durations were again prolonged, maintaining a similar level to cells at normal conditions. By contrast, the average OFF-time of rDNA transcription did not change significantly at different culture conditions. In addition to ON-time modulation, burst amplitude was slightly increased in response to growth stimulation (Fig. 7, F and G). Notably, both rDNA loci we have monitored exhibited consistent phenotypes. Thus, our results suggest that rDNA transcriptional response to starvation or growth stimulation is achieved by the modulation of burst duration (ON-times) and burst amplitude.

### Investigating Pol I regulators through LiveArt

**Figure 8.**
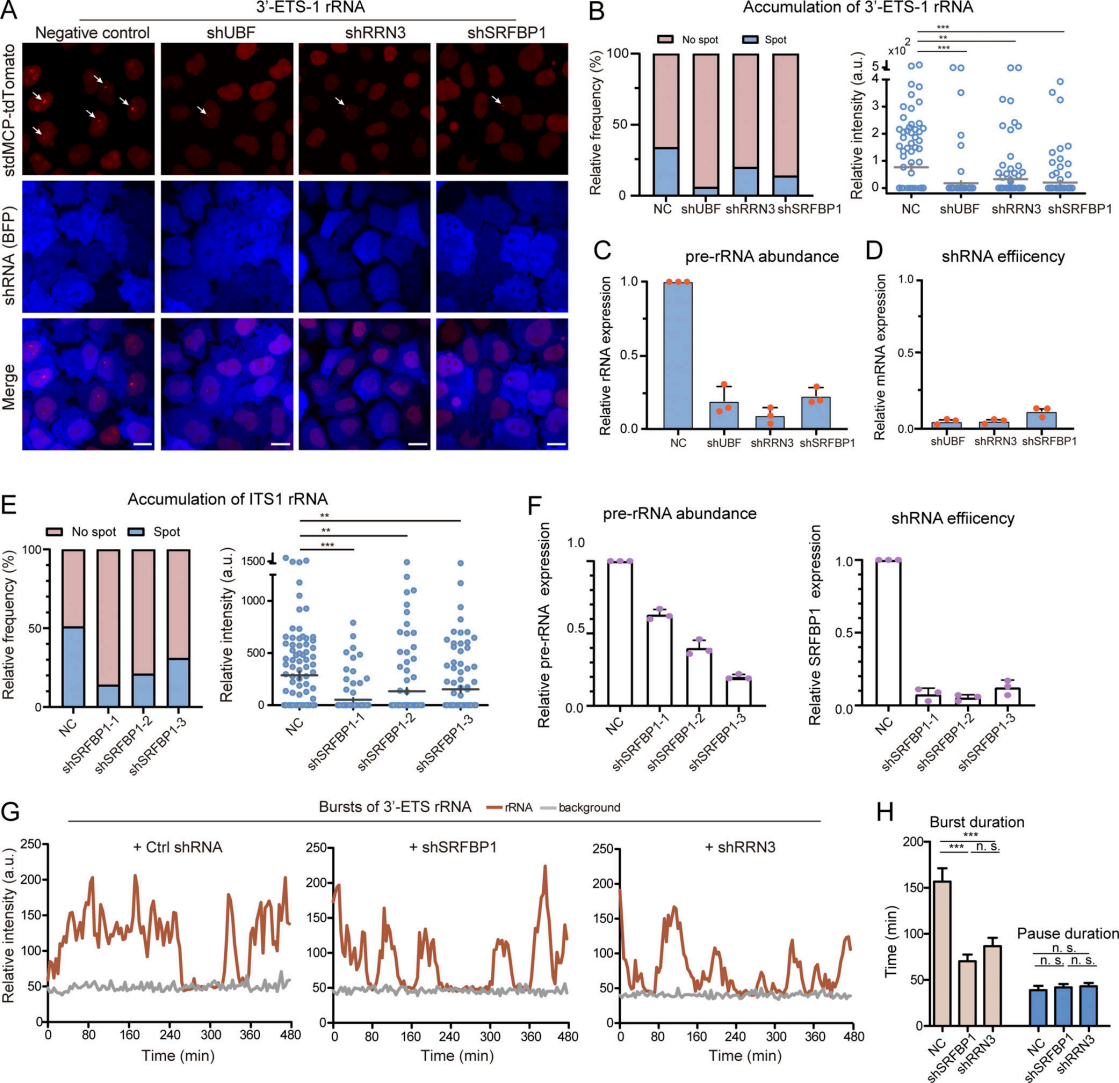
**Investigation of Pol I transcriptional control using LiveArt. (A)** Representative images illustrating the changes of 3′-ETS-1 rRNA when UBF, RRN-3, or SRFBP1 was downregulated by shRNA in clonal cells. BFP protein was imaged to indicate shRNA expression. All images are maximum-intensity projections from *z* stacks. Scale bars, 10 µm. Arrows point to visible MS2-tagged rRNAs. **(B)** Left: Bar graph showing the proportion of cells with visible stdMCP-tdTomato spots representing 3′-ETS-1 rRNAs under various conditions in A (*n* ≥ 178). Right: Quantification of 3′-ETS-1 rRNAs accumulation in each cell by measuring total intensity of individual stdMCP-tdTomato spot. Each dot represents a single cell (*n* = 100). Gray line indicates mean ± SEM. **(C and D)** Measurement of 45S pre-rRNA abundance (C) or shRNA efficiency (D) by qPCR. *n* = three biological replicates displayed as mean ± SEM. **(E)** Left: Bar graph showing the proportion of cells with visible ITS1 rRNA indicated by stdMCP-tdTomato under different conditions (*n* ≥ 154). Right: Quantification of ITS1 rRNA accumulation in each cell by measuring total intensity of individual stdMCP-tdTomato spot. Each dot represents a single cell (*n* = 100). Gray line indicates mean ± SEM. **(F)** qPCR to examine relative expression of 45S-preRNA (left) and SRFBP1 (right) in SRFBP1 down-regulated samples from E. *n* = 3 technical replicates. **(G)** Representative traces (red) demonstrating the real-time synthesis of MS2-tagged rRNAs (3′-ETS-1) by measuring the maximum intensity of stdMCP-tdTomato spots in corresponding cells that were infected with negative control (NC) shRNA or shRNAs to specifically down-regulate SRFPB1 and RRN3, respectively. **(H)** Quantification of burst duration and pause duration by imaging 3′-ETS-1 clone. *n* ≥ 100. Data are all displayed as mean ± SEM. One-way ANOVA with Tukey’s post hoc was used to test differences between groups. ***P ≤ 0.001, **P ≤ 0.01. n.s., not significant.

LiveArt allows quantification of rRNA synthesis in real time. We therefore assessed the capability of LiveArt for elucidating the regulatory role of nucleolar factors. We first performed small hairpin RNA (shRNA)–mediated knockdown of UBF (upstream binding factor) and RRN-3, which are known to be activators of Pol I transcription (Bell et al., 1988; Hein et al., 2013; Jantzen et al., 1990; Learned et al., 1986). As expected, the percentage of cells with visible 3′-ETS rRNA foci was decreased (Control, 34%; UBF, 6%; RRN-3, 20%), and the accumulation of 3′-ETS rRNAs was significantly repressed (UBF, 4.4-fold; RRN-3, 2.4-fold) when Pol I transcription activators were repressed (Fig. 8, A and B). These results document the effectiveness of LiveArt for the detection of rRNA synthesis.

Then, we performed a candidate-based RNA interference screen of nucleolar proteins that effectively affect rDNA transcription by implementing LiveArt. We identified serum response factor binding protein 1 (SRFBP1, also named p49/STRAP) as a potential regulator of rRNA synthesis. SRFBP1 knockdown repressed 3′-ETS rRNA production by a factor of 3.7 (Fig. 8 B). shRNA efficiency and the effects on rDNA transcription were confirmed by quantitative RT-PCR (qRT-PCR; Fig. 8, C and D). We also carried out experiments using ITS1 clone and designed two more shRNAs targeting SRFBP1, and observed similar results (Fig. 8, E and F). To further investigate how SRFBP1 affects rRNA synthesis, we quantified the transcriptional kinetics of rDNA in SRFBP1-depleted cells mediated by shRNA. The duration of on state was decreased from 156.8 to 70.3 min, whereas the off state duration remained nearly unchanged (38.9 vs. 41.9 min). Similarly, as a control, downregulation of RRN3 by shRNA affects the durations of on state, but not off state (Fig. 8, G and H). Our results suggest that SRFBP1 may play a role in the regulation of rDNA transcription.

**Figure 9.**
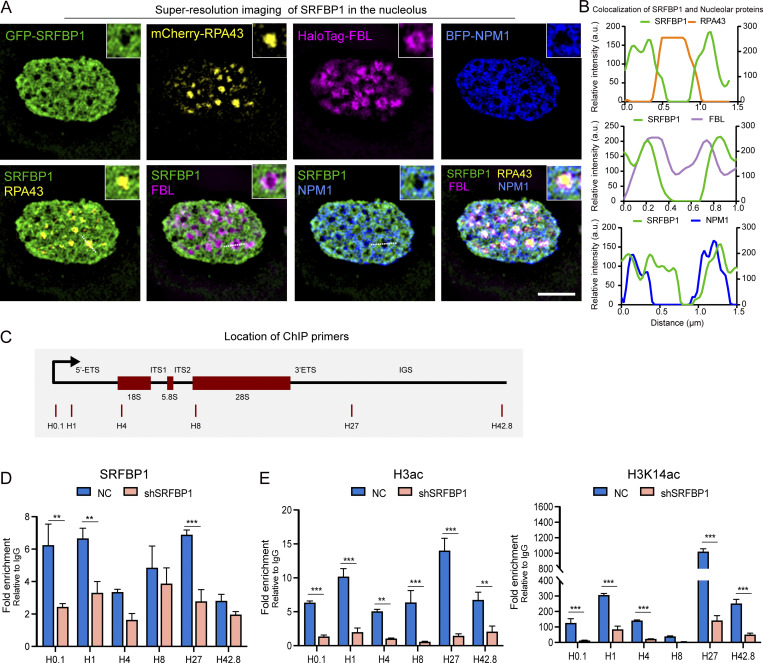
**SRFBP1 is involved in Pol I regulation. (A)** SIM imaging showing the co-localization of SRFBP1 with nucleolar markers in HeLa cells. FC: RPA43, DFC: FBL, GC: NPM1. Scale bar, 2 μm. Insert magnification: 2.7×. **(B)** Line scan of the relative fluorescence of the signal indicated by the dotted lines in A. **(C)** Relative position of the ChIP primers on the rDNA locus. **(D and E)** Bar plots showing ChIP-qPCR analysis of the relative enrichment of SRFBP1 (D), H3ac, or H3K14ac (E) at rDNA loci in control (NC) and SRFBP1 depleted Hela cells. Data are display as mean ± SEM. *n* = three technical repeats. Multiple *t* test, **P ≤ 0.01, *** P ≤ 0.001.

SRFBP1 is previously suggested to be a cofactor of SRF (serum response factor), contributing to the transcriptional regulation of cardiac genes during aging (Zhang et al., 2004). Intriguingly, SRFBP1 is highly enriched in the nucleoli (Lin et al., 2009; Zhang et al., 2014b). We applied Hessian-SIM to image the subcellular localization of GFP-SRFBP1 at high spatial resolution and observed that SRFBP1 closely surrounded RPA43-labeled FCs, which is known to be a subcompartment harboring active rDNA repeats. Additionally, SRFBP1 is also enriched in DFCs and GCs (Fig. 9, A and B). Moreover, we used chromatin immunoprecipitation (ChIP) assays with anti-SRFBP1 to determine the presence of SRFBP1 along the rDNA repeat. qPCR quantifications used sets of primer pairs spanning the entire rDNA repeat (Grandori et al., 2005). Our results revealed that SRFBP1 may directly or indirectly bind to rDNA, including the transcriptional start site (Fig. 9, C and D). We then assessed whether depletion of SRFBP1 affects histone modifications of rDNA repeats. Previous studies suggest that histone H3 acetylation is related to active genes, including rDNA with open states (Koch et al., 2007; Plata et al., 2009; Wang et al., 2008). Thus, we used ChIP-qPCR to measure the acetylation levels of histone H3 (H3ac) and histone H3 at 14 lysine position (H3K14ac). Our results demonstrated that the levels of H3 acetylation at the rDNA locus were significantly reduced in SRFBP1-depleted cells (Fig. 9 E). Of note, SRFBP1 was also identified as an rRNA processing factor in a previous screening using northern blot analysis (Tafforeau et al., 2013). Therefore, how SRFBP1 regulates rRNA biogenesis needs to be further addressed in future.

### Application of LiveArt for anticancer drug screening

**Figure 10.**
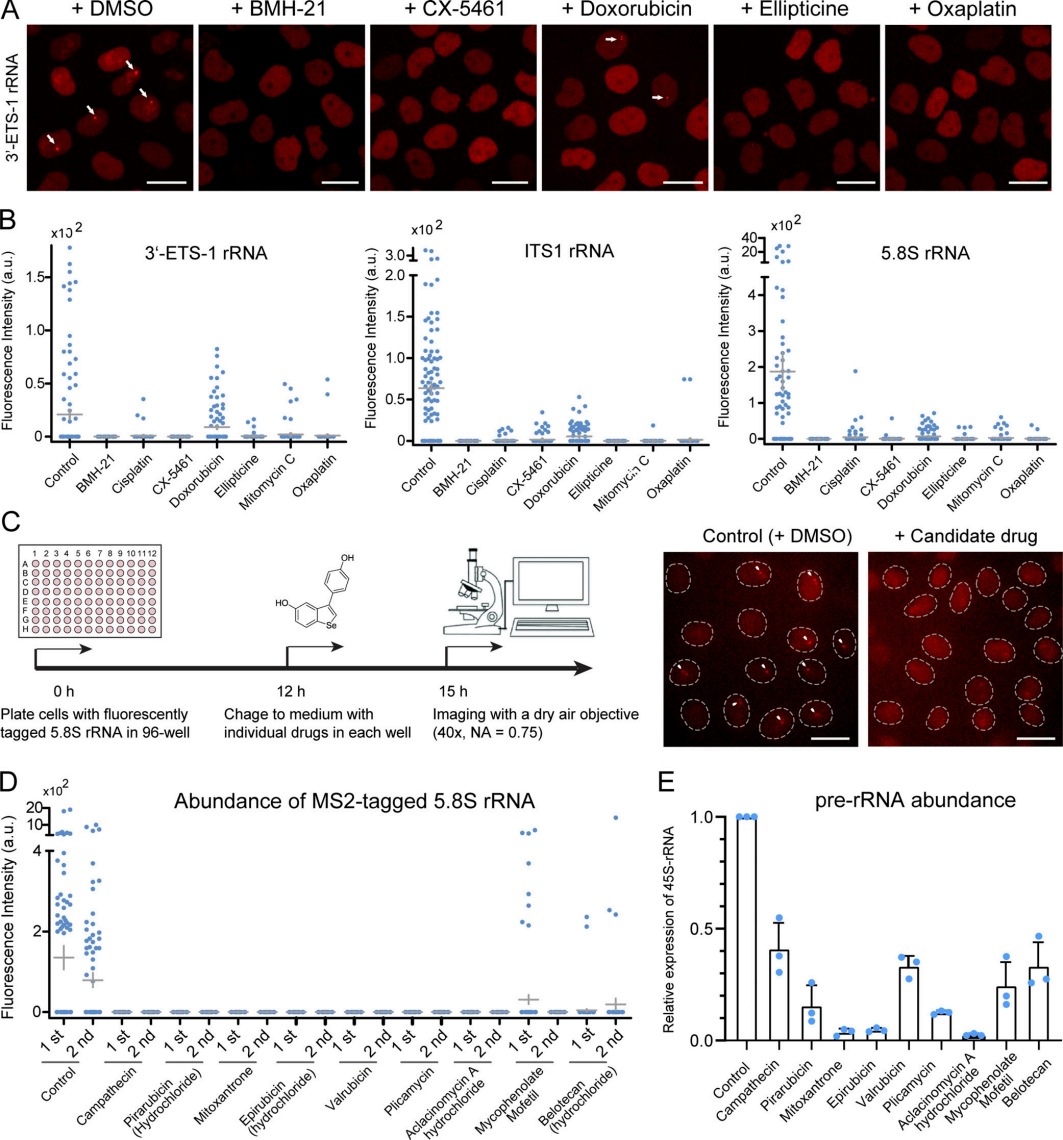
**LiveArt acts as a robust reporter system for anti-cancer drug screening. (A)** Representative images to show the effect of anti-cancer drugs on rRNA synthesis in clonal cells with MS2-tagged 3′-ETS-1 rRNA. Arrows point to the enriched signal of stdMCP-tdTomato. All images are maximum-intensity projections from *z* stacks. Scale bars, 10 µm. **(B)** Quantification of total MS2-tagged 3′-ETS-1 rRNA (left), ITS1 rRNA (middle), or 5.8S rRNA (right) produced per nucleus in the absence or presence of individual anti-cancer drugs. Each dot represents a single cell (*n* = 100). Gray line indicates mean ± SEM. **(C)** Workflow used to perform anti-cancer drug screening via LiveArt. **(D)** Statistics of MS2-tagged 5.8S rRNA accumulation revealed by the total intensity of stdMCP-tdTomato spots in the absence or presence of anti-cancer drugs, which were screened out from the library of FDA-approved drugs. Each dot represents a single cell (*n* = 100). Gray line indicates mean ± SEM. **(E)** Measurement of 45S pre-rRNA abundance. *n* = three technical replicates displayed as mean ± SEM.

Since rDNA transcription has been recognized as a potential effective target for cancer therapy, we asked whether LiveArt could serve as a new platform for drug screening. First, we tested seven available anticancer drugs that are known to exhibit their therapeutic potential by repressing Pol I transcription, including CX-5461, BMH-21, Ellipticine, Cisplatin, Doxorubicin, Oxaplatin, and Mitomycin C (Ferreira et al., 2020; Hein et al., 2013). Based on imaging, LiveArt systems indicated that all seven drugs show significant repression of rRNA synthesis with quick response within 3 h of drug incubation (Fig. 10, A and B). CX-5461, as the first direct and selective inhibitor of RNA Pol I, holds great promise for cancer therapeutics (Drygin et al., 2011; Haddach et al., 2012; Khot et al., 2019). Indeed, CX-5461 treatment resulted in dramatic inhibition on rRNA production. For example, the mean intensity of 5.8S was dropped from 187.3 to 0.7 (Fig. 10 B).

To further assess if LiveArt could serve as an efficient platform for drug screening, we performed a pilot screen, including 2374 FDA-approved drugs (Fig. 10 C). By performing quantitative rRNA imaging using 5.8S clone, we identified 26 hit candidates which turn out to be drugs that are currently being used for cancer research or therapy. We quantified nine of them by both imaging and qRT-PCR. These drugs robustly repressed rRNA synthesis within 3 h (Fig. 10, D and E). Our results suggest that these anticancer drugs exert, at least partially, their activity through disruption of Pol I transcription. How these drugs are involved in the rRNA synthesis pathway need to be further clarified. In conclusion, LiveArt demonstrates excellent sensitivity, quick response, and high reproducibility for monitoring Pol I transcription, facilitating a reliable and rapid assay for cell-based screening of anticancer drugs.

## Discussion

In this study, we have developed a new rRNA labeling method, LiveArt, by which the transcriptional kinetics of rDNA mediated by Pol I can be monitored in living cells. RNA labeling technologies have been widely developed for the quantitative imaging of Pol II activity by inserting MS2 (or other aptamers) into the intron or UTR region of target genes (Chubb et al., 2006; Fritzsch et al., 2018; Larson et al., 2013; Lee et al., 2019; Muramoto et al., 2012). However, Pol I transcriptional activity has not been visualized in real time. Our results indicate that MS2 labeling strategy can also be applied to visualize rRNAs. First, MS2-tagged rRNAs were specifically produced in the nucleoli and their production can be validated by FISH and effectively blocked by Pol I inhibitors (ActD or CX-5461). Second, the transcriptional bursting of Pol I can be visualized by tagging 3′-ETS because the ETS region can be rapidly cleaved once transcribed. In contrast, 5.8S rRNAs accumulate at different sites in the nucleolus, representing the behavior of both nascent and mature rRNAs. Third, LiveArt can report the process of switching rDNA transcription off and on during mitosis. Fourth, both down- and up-regulation of Pol I transcription can be detected through LiveArt. Collectively, we conclude that the LiveArt technology can reflect the transcriptional activity of Pol I in real time.

The currently available rRNA probes, such as the use of J1 or ECHO (Cao et al., 2019; Du et al., 2017; Oomoto et al., 2015), make it possible to detect bulk native rRNAs without genetic engineering in living cells. General limitations of these probes are the transiently labeling due to the trapping of probes by endocytic pathway, and the low targeting specificity (Sato et al., 2020). Compared to these methods, there are two significant advantages of LiveArt: (1) Only rRNAs generated from single or low copy number of rDNA units are fluorescently labeled in the nucleoli. (2) Stable tagging of rRNAs can be maintained by isolating clonal cells. Because of these advantages, we can observe the transcriptional kinetics of Pol I under various cellular conditions and monitor the spatiotemporal dynamics of rRNAs over cell divisions by long-term live imaging. Moreover, the LiveArt clonal cell line provides a simple but powerful platform for large-scale screening of Pol regulators. Therefore, our method offers complementary benefits to other rRNA detection techniques. Importantly, the use of these different methods allows for better validation of results related to Pol I regulation.

The development of LiveArt benefits from advances in the CRISPR-Cas technology (Chen et al., 2020; Knott and Doudna, 2018; Suzuki et al., 2016). To achieve single or low copy number of MS2 insertion in rDNA, CRISPR editing efficiency can be minimized by reducing the amount of CRISPR plasmids transfected into the cells or using RNP delivery to limit the duration of Cas9/sgRNA expression (Lin et al., 2014). A cleavage-free gene editor, dCas9-SSAP, is recently developed for efficient long-sequence knockin with low on-target errors and minimal off-target effects in mammalian cells (Wang et al., 2022). This strategy is a theoretically ideal editing tool for creating LiveArt clonal cells. Of note, clonal isolation of rRNA tagging cells is a crucial step of implementing LiveArt. A careful analysis of MS2 insertion should be performed to validate LiveArt clonal cells. These validations will shed light on how to utilize the clonal cells for analyzing Pol I transcription. A clonal cell line with single-rDNA tagging is particularly suitable for measuring Pol I transcriptional bursting. In addition, regardless of the copy number of rDNA tagging, clonal cells with bright rRNA signals can be potentially used to report Pol I transcription in real time.

Our functional analyses (including ribosome biogenesis, protein translation, and cell proliferation) suggest that LiveArt could monitor rRNA synthesis with minimal impact on the function of bulk rRNAs and cellular behavior. However, it is technically challenging to compare RNA folding, processing and stability between MS2-tagged and untagged rRNAs. Moreover, recombinant rRNAs with MS2 insertion in the 5.8S region may not assemble into mature ribosomes. Therefore, whether the current LiveArt system is suitable for analyzing rRNA processing and other downstream events requires further investigation. Future optimizations are needed to minimize these potential effects, possibly through reducing the size of MS2 cassette and utilizing other RNA aptamers (Cawte et al., 2020; Daigle and Ellenberg, 2007; Das et al., 2021; Filonov et al., 2014; Larson et al., 2011). Additionally, it is important to note that transcription and RNA processing are two interdependent events (Neugebauer, 2019). Under certain conditions, the aberrant accumulation of fluorescently labeled RNAs may be contributed by the defects in the RNA processing pathways. Thus, a combination of analyses (e.g., rRNA imaging, qPCR, or Northern blot) should be performed to fully address the causes of altered rRNA accumulation.

Our results demonstrate that LiveArt can detect the transcriptional dynamics of Pol I in living cells by 3′-ETS tagging. Our live imaging data indicate that rDNA is transcribed in long bursts alternated by periods of silence where no or very little rRNA is being produced. rDNA expression levels can be adjusted by altering the duration time and amplitude of bursts. Moreover, the kinetics of rRNA synthesis can now be coupled with single-cell imaging of other nucleolar components (rDNA and nucleolar proteins). Our live imaging results demonstrate the importance of rRNA for nucleolar formation and maintenance, which is consistent with other findings based on in vitro assays (Feric et al., 2016; Mitrea et al., 2016; Mitrea et al., 2018). Notably, our approach has provided more detailed dynamic information on nucleolar dynamics. Increasing evidence provided strong support for the formation of nucleoli as phase-separated condensates (Lafontaine et al., 2021). It is suggested that RNA turns the fluidity of various phase-separated condensates (Boeynaems et al., 2019; Zhang et al., 2015). Thus, LiveArt will provide temporal insights into nucleolar architecture and functions. Moreover, we anticipate that, through direct visualization of the rRNA production in living cells, LiveArt can provide an efficient platform for high-throughput screens for novel drugs to combat cancer through down-regulation of Pol I transcription. Taken together, LiveArt technology appears to be useful for both basic research and drug discovery.

## Materials and methods

### Cell culture

HEK293T cells and HeLa cells were cultured in DMEM with high glucose (Gibco) in 10% FBS (Hyclone) and 1% penicillin/streptomycin (Gibco). U2OS cells were grown in McCoy’s 5A (Procell) supplemented with 10% FBS, 1% penicillin and streptomycin. RPE-1 cells were cultured in DMEM/F-12 (Hyclone) supplemented with 10% FBS, 1% penicillin and streptomycin. All cells were cultured at 37°C and in a humidified incubator with 5% CO_2_. Cells used in this study were maintained in mycoplasma-free status.

### Plasmid construction

The Addgene plasmids #40649 (Wu et al., 2012) and #164044 (Xu et al., 2020) were used to express tdMCP-GFP and stdMCP-tdTomato, respectively. The construction of dCas9-GFP11_14X_ and GFP1–10 plasmids has been described in our previous study (Chen et al., 2018). The following plasmids were specifically constructed for this study.

#### Construction of donor plasmids

To build plasmids for live-cell RNA labeling, the DNA fragment 17xMS2V5 was amplified from an Addgene plasmid #84561 (Wu et al., 2016). To insert MS2V5 into different rDNA regions, we performed CRISPR/Cas9-based HITI (Suzuki et al., 2016). To construct the donor plasmid, the DNA fragment of 17xMS2V5 harboring Cas9 recognized cleavage sequences (5′-GCA​CCG​ATG​CTC​TCC​GAG​GAGG-3′, named TS3; the PAM sequence is underlined) at both ends was cloned into a small vector using T4 DNA ligase (New England Biolabs). This plasmid was used as a universal donor plasmid for all labelings of rRNAs in Fig. S1, including 5′-ETS, 18S, ITS1, 5.8S, ITS2, 28S, and 3′-ETS. The sgRNA recognizing TS3 can cleave donor plasmid, thus release a linear fragment of MS2V5 for higher knockin efficiency.

#### Construction of sgRNA plasmids

All the sgRNAs used in this study were constructed by modifying a sgRNA vector (#164043; Addgene; Chen et al., 2018). The spacer sequence determining the target sequence can be changed to recognize a new site by the PCR-based QuikChange cloning method. To construct sgRNA for labeling rDNA, the Golden Gate Cloning method was used to assemble multiple sgRNAs (sgrDNA-1, sgrDNA-2, sgrDNA-3, and sgrDNA-4) into CRISPRainbow-donor vector (#75398; AddGene; Ma et al., 2016). All sgRNAs used in this study are listed in Table S1.

#### Construction of reporter plasmids

To construct plasmids for expressing GFP-RPA43, mCherry-RPA43, HaloTag-FBL, BFP-NPM1, H2B-GFP, or GFP-SRFBP1, the full-length of RPA43, FBL, NPM1, H2B, and SRFPB1 were amplified individually from human cDNAs (prepared using HeLa cells) and inserted into our lentiviral backbone vector pHR-SFFV (same backbone with Addgene #80409; Kamiyama et al., 2016), respectively, using NEBuilder HiFi DNA Assembly Cloning Kit (New England Biolabs).

#### Construction of shRNA plasmids

To construct plasmids for expressing shRNAs, we modified an Addgene plasmid pLKO.1-TRC vector (#10878; Moffat et al., 2006). We first replaced Puromycin with BFP using T4 ligase. Oligos, harboring the shRNA sequence flanked by sequences that are compatible with the sticky ends of EcoRI and AgeI, are annealed and ligated into pLKO.1-TRC vector through T4 ligase, producing a final plasmid that expresses the shRNA of interest. shUBF and shSRFBP1 used in this study were validated in a previous study (Tafforeau et al., 2013). All shRNA targeting sequences are listed in Table S2.

### Lentivirus production and generation of clonal cell lines

To produce lentivirus, HEK293T cells were seeded into 12-well plates. After ∼12 h, cells were transiently transfected with 750 ng lentivirus constructs (dCas9-GFP11_14X_, GFP1–10, stdMCP-tdTomato, tdMCP-GFP, GFP-RPA43, mCherry-RPA43, HaloTag-FBL, BFP-NPM1, GFP-SRFBP1, or H2B-BFP), 705 ng pCMV-dR8.91, and 87 ng PMD2.G, using polyethylenimine transfection reagent (Polysciences) following the manufacturer’s recommended protocol. Virus was harvested 60 h after transfection, centrifuged at 800 *g* for 8 min to collect supernatant, and directly added to cells or frozen at –80°C. To produce concentrated lentivirus for shRNA expression, HEK293T cells were seeded into T25 flask. After ∼12 h, cells were transiently transfected with 2,500 ng shRNA lentiviral constructs, 1,250 ng psPAX2, and 625 ng PMD2.G, using polyethylenimine following the manufacturer’s recommended protocol. Concentrated shRNA lentiviruses were obtained by precipitating viral containing supernatants with PEG6000 (Solarbio). HeLa cells were infected with tdMCP-GFP or stdMCP-tdTomato lentivirus for live-cell rRNA labeling. RPE-1 and U2OS cells were also infected with stdMCP-tdTomato lentiviruses for rRNA labeling. To achieve simultaneous labeling of rDNA and rRNA, HeLa cells were infected with dCas9-GFP11_14X_, GFP1-10, and stdMCP-tdTomato lentiviruses. To enhance the infection of each lentivirus, cells were infected in the presence of polybrene (5 µg/ml). HeLa, U2OS, RPE-1 clonal cell lines that express these components at an optimal level for the best labeling of rRNA or rDNA were isolated and used for imaging experiments. These clonal cell lines were selected based on the signal-to-noise ratio of rRNA or rDNA labeling.

### CRISPR-mediated knockin

To label rRNAs transiently, HeLa cells expressing stdMCP-tdTomato (or tdMCP-GFP) were grown in 8-well chambered coverglass and transiently transfected with Cas9 (plasmid #64323 or #62988; Addgene; Chu et al., 2015; Ran et al., 2013), donor, sgrDNA (sgRNA targeting rDNA), and sgTS3 (sgRNA targeting TS3 sequence) plasmids. The optimal amount of each plasmid is: 2.5 ng Cas9, 200 ng sgTS3 (for generating double-cut donor), 5 ng donor, and 5 ng sgRNA expression vectors targeting most rDNA regions. sgTS3 was used to cleave donor plasmid and release the fragment of 17XMS2V5, thus enabling the integration of MS2 sequence into a specific rDNA region through CRISPR-Cas9 mediated HITI. To label 3′-ETS rRNA in U2OS or RPE-1 cells, same amounts of Cas9, sgRNA, and donor were transfected. Notably, MS2 knockin should always be tested and optimized for labeling a specific region of rRNAs in a particular cell type. The knock-in procedure can be serially titrating concentrations of Cas9, sgRNA, and donor constructs. The donor sequence for HITI is shown in Table S3. To insert 17XMS2V5 by HDR, the donor was prepared by PCR which amplified 5′ HA (39 bp)-17XMS2V5-3′ HA (38 bp), which is shown in Table S4. HDR efficiency is generally lower than HITI for rRNA tagging. The amount of each plasmid used for HDR in HeLa (8-well) is: 2.5 ng Cas9, 50 ng donor, and 50 ng sgRNA expression vectors targeting most rDNA regions.

### Generation of HeLa clonal cell lines with stable rRNA tagging

To achieve single or low copy number of rDNA tagging by MS2, we kept CRISPR editing efficiency (both HITI and HDR) below 2%. Thus, we could only detect rRNA synthesis in about 1–2% of cells in the pool. The HITI strategy was used as an example to describe the procedures for isolating clonal cells with stable rRNA labeling. Hela cells expressing particular reporters were seeded on 24-well plates and transiently transfected with 5 ng Cas9 protein-expressing vectors, 20 ng donor plasmids, 20 ng sgrDNA (targeting 3′-ETS, ITS1, or 5.8S of rDNA), and 200 ng sgTS3 (targeting donor plasmid) expression vectors. HeLa clonal cell lines with stable rRNA labeling were isolated from pooled knock-in cells. Because these clonal cell lines were selected based on the rRNA labeling, they were referred to rRNA clonal cells hereafter. Notably, there are two ways to isolate the clonal cells from the pooled MS2 knock-in cells (CRISPR editing rate could be as low as ∼ 1.5%). One strategy is to directly establish single-cell clonal cultures in 96-well plates by limiting dilution. The positive rate of obtained clonal cells with stable rRNA tagging is around 0.5% (our case). That is, it is likely to isolate one positive clone by screening ∼200 clones. Another strategy is to suspend the pooled MS2 knock-in cells and seed ∼10 cells into each well in 96-well plate. The rate to identify one well-containing rRNA tagging cells is around 1.4%. That is, it is possible to find out one well containing positive cells by screening ∼70 wells. Clonal cells could be further isolated from this well and the positive rate is around 10% theoretically (our case is ∼ 4%). We only isolated HeLa clonal cells for rRNA tagging. Other cell types might need to be further optimized.

### Copy number of MS2 cassette

To analyze the copy number of MS2 cassette (17xMS2V5) in rRNA clonal cells, we first determined the copy number of *MUC4* by CRISPR imaging, which was previously developed by us (Chen et al., 2013). Quantitative results clearly indicated that all the four clonal cell lines contain three *MUC4* loci. Therefore, *MUC4* was selected as the reference gene. Fragments of 17xMS2V5 and *MUC4* were cloned into a vector. Following a published method (Johnson et al., 2015), we generated standard curves with dilutions of p*MS2V5*_*17X*_ and p*MUC4* plasmids, each ranging between 10^3^ and 10^7^ copies/5 µl. qPCR was performed using ChamQ Universal SYBR qPCR Master mix (Vazyme) on the CFX96 Real-Time PCR system (Bio-Rad). The copy numbers were calculated according to the standard curve and the Ct values using genomic DNA as the qPCR template. qPCR primers are listed in Table S5.

### rDNA labeling

To achieve the best signal-to-noise ratio of rDNA labeling, a clonal cell line stably expressing dCas9-GFP_14X_ was selected for CRISPR imaging, which has been described in our previous studies (Chen et al., 2018). To examine the colocalization between rDNA and rRNA, HeLa cells stably expressing dCas9-GFP_14X_ and stdMCP-tdTomato were plated into 8-well chambered coverglass (Lab-Tek II) and transfected with a single sgRNA plasmid (600 ng). This sgRNA plasmid could express four sgRNAs that recognize different genomic regions of rDNA to enhance the labeling.

### In situ hybridization to MS2-tagged rRNAs

A series of short probes (Cat # 2903461-D1) complementary to MS2V5_17X_ sequence, covering the entire region of MS2V5_17X,_ were designed and synthesized by GD Pinpoease Biotech Co., Ltd. The RNA in situ hybridization was performed using PinpoRNATM RNA in situ hybridization kit (Cat # PIT1000; GD Pinpoease Biotech Co. Ltd.) according to the manufacturer’s instruction. Briefly, the cells were fixed by 10% neutral-buffered formalin and then the endogenous peroxidase was inhibited by Pre-A solution at room temperature. Target RNA molecules were exposed by protease treatment and hybridized with probes for 2 h at 4°C. Then the signal was amplified sequentially by reactions 1, 2, and 3. The HRP molecule was added into reaction 3. Finally, a tyramide fluorescent substrate (OpalTM520, Akoya Biosciences) was added and the target RNA was thus labeled by green fluorescence by tyramide signal amplification assay.

### Serum stimulation

rRNA clonal cells were seeded into 8-well chambered coverglass (Cellvis) with 10% FBS. The next day, cells were maintained in serum-free condition. After being starved for 24 h, cell culture was switched to the normal state with 10% FBS, which initiated serum stimulation. Quantitative imaging was performed 30 min after serum stimulation. To analyze transcriptional bursting, cells that have been starved for 24 h were transferred to a humidified chamber maintained at 37°C and 5% CO_2_ and monitored by real-time imaging for 8 h without or with the addition of 10% FBS.

### Immunostaining

Cells were fixed in 4% paraformaldehyde, permeabilized with 0.5% NP-40 in PBS for 10 min, washed with PBS for 5 min, blocked in 0.2% cold water fish gelatin and 0.5% BSA for 20 min, incubated with the primary antibody in blocking buffer at 4°C overnight, washed three times, and then incubated with Alexa647-conjugated secondary antibody at room temperature for 1 h, and washed three times again. Primary antibodies used in this study are anti-phospho-Histon H2A.X (#05-636-I; EMD Millipore) and anti-CC3 (Asp175). Secondary antibody is Alexa Fluor 647–labeled Goat Anti-Mouse IgG H&L (ab150115; Abcam). Hoechst 33342 dye is utilized as a nuclear stain in the immunofluorescence if necessary.

### Northern blot

To detect pre-rRNA processing, 10 µg total RNA was separated by electrophoresis on 1% agarose/2% formaldehyde gel and transferred to a positively charged nylon membrane by capillary transfer. After UV-crosslink, the hybridization was performed with Digoxin-labeled DNA probes specific for ITS1 region. Blots were detected using Digoxin Northern Starter Kit from Roche Applied Science following the manufacturer’s instruction. The probe sequence is 5′-AAG​GGG​TCT​TTA​AAC​CTC​CGC​GCC-3′.

### Puromycin incorporation

To measure global protein synthesis, cells were incubated with puromycin (1 µM final concentration) for 30 min at 37°C and 5% CO_2_. Cells were then harvested by centrifuging at 4°C. Total protein extracts (15 µg for each sample) were run on SDS-polyacrylamide gels, transferred to polyvinylidene difluoride membranes followed by incubation with anti-puromycin antibody (Kerafast-EQ0001, 1 µg/ml in Tris-buffered saline containing Tween-20) overnight at 4°C, goat anti-mouse HRP secondary antibody (HuaBio; 1:5,000) at room temperature for 1 h, and finally developed with ECL reagents following manufacturer’s instructions.

### Polysome profiling

Polysome profiling was conducted as previously described (Pringle et al., 2019). In brief, prior to harvesting, cells were treated with 100 µg/ml cycloheximide (CHX) for 3 min at 37°C. Cells were washed twice with ice-cold CHX/PBS, gently scraped from the plate and resuspended in lysis buffer (10 mM Hepes, pH 7.4, 5 mM MgCl_2_, 100 mM KCl, 1% Triton X-100, 1 mM DTT, 100 U/ml RNase inhibitor, protease inhibitor cocktail, 1 mM PMSF, 100 µg/ml CHX) and then incubated on ice for 15 min. The cytoplasmic fraction was extracted by low-speed centrifugation and loaded onto a 10–50% sucrose gradient. The sucrose gradients were centrifuged in a SW41Ti rotor (Beckman) at 38,000 rpm for 2.5 h. 10 fractions (∼1 ml each) were collected using a Piston Gradient Fractionator (Biocomp). Polysome profiles were recorded at 260 nm using a Gradient Profiling Instrument (Biocomp).

### Isolation of ribosomes

Ribosomes were isolated with a previously described protocol (Belin et al., 2010). In brief, cells were collected and lysed with 250 μl cold lysis buffer (10 mM Hepes, pH 7.4, 5 mM MgCl_2_, 100 mM KCl, 1 mM DTT, 1% Triton X-100, protease inhibitor cocktail, 1 mM PMSF), incubated on ice for 10 min. Cell lysates were centrifuged for 10 min at 12,000 *g*, 4°C. The supernatant was transferred into a new tube and the nuclear pellet was collected for further Western blot analysis. 200 μl of supernatant was added above 800 μl sucrose cushion (1 M sucrose, 10 mM Hepes, pH 7.4, 5 mM MgCl_2_, 100 mM KCl, protease inhibitor cocktail, 1 mM PMSF) and ultra-centrifuged at 120,000 rpm, 4°C for 2.25 h in an MLA150 rotor (Beckman). The ribosomal fraction was then washed and collected for rRNA purification. Western blot was performed to check the purity of ribosome fraction using the following primary antibody: anti-RPL22 rabbit polyclonal (Proteintech, 25002-1-AP) and anti-NPM1 mouse monoclonal (Santa Cruz, FC82291).

### ChIP assay

ChIP assay was performed with a ChIP assay kit (#17-371; Millipore) according to the manufacturer’s instructions. Briefly, chromatin DNA was crosslinked by adding formaldehyde directly into the media at a final concentration of 1% and incubated at room temperature for 10 min. Crosslinking reaction was stopped by adding excessive glycine. The cells were washed, scraped, pelleted, and lastly lysed in SDS lysis buffer. Crosslinked DNA was sheared by sonication. Chromatin was diluted 10-fold with ChIP dilution buffer. Chromatin was precipitated with their respective antibodies. IP was performed using 3 μg of rabbit polyclonal anti-acetyl-Histone H3 (#07-353; Sigma-Aldrich), anti-acetyl-Histone H3 (Lys14; #07-353; Sigma-Aldrich) or anti-SRFBP1 (ab109598; Abcam) antibodies, and 1 μg of rabbit IgG was used as negative control. Incubation was performed overnight at 4°C with rotation. To collect the antibody/antigen/DNA complex, 60 μl Protein G Agarose was added to each IP and incubated for 1 h at 4°C with rotation. Enriched chromatin DNA was then purified for further qRT-PCR. Primers for qRT-PCR were reported (Grandori et al., 2005) or designed and listed in Table S6.

### Colony formation assay

At the exponential growth phase, cells were harvested with trypsin-EDTA and counted using a hemocytometer. Following this, cells were diluted and seeded at about 1,500 cells per well of a 6-well plate. After incubation for 5 d, cells were washed with PBS twice, fixed with 4% paraformaldehyde for 15 min, and stained with Hoechst 33342 for 15 min at room temperature. Cell number in each clone was calculated based on fluorescent imaging.

### Drug treatment

To validate the labeling of rRNAs, we took advantage of ActD (MCE, HY-17559), which selectively inhibits transcription activity of RNA Pol I polymerase at a low concentration (25 ng/ml). However, 12.5 ng/ml ActD was sufficient to suppress rDNA reactivation during mitosis. To block rRNA processing, cells were treated with 10 µM 5-FU (MCE-HY90006) for 24 h. To further validate the use of LiveArt for anticancer drug screening, available drugs, including BMH-21 (MCE, HY-12484), Cisplatin (MCE, HY-17394), CX-5461 (MCE, HY-13323; TargetMol, T2100), Doxorubicin (hydrochloride; MCE, HY-15142), Ellipticine (hydrochloride; MCE, HY-15753A), Mitomycin C (MCE, HY-13316), Oxaplatin (MCE, HY-17371) were tested in rRNA clonal cells. Cells with appropriate reporters were seeded into 8-well chambered coverglass (Cellvis) prior to drug treatments. The next day, cells were recorded using a spinning-disk confocal microscope or widefield microscope in the absence or presence of drugs. Final concentration of BMH-21, Cisplatin, CX-5461, Doxorubicin, Ellipticine, Oxaplatin, and Mitomycin C were 1 µM, 100 µM, 1 µM, 1 µM, 10 µM, 100 µM and 100 µg/ml, respectively. To induce apoptosis, ITS1 rRNA clonal cells were treated with 1 µM RITA (TOPSCIENCE, T1798) for 3 d before CC3 staining.

### A pilot screen of anti-cancer drugs

An FDA-approved drug library containing 2,374 compounds (MCE, HY-L022) was screened using LiveArt. All compounds were diluted to 10 µM as the final concentration. Clonal cells with stable 5.8S rRNA tagging were seeded in a 96-well plate prior to drug treatment. After 3 h of drug treatment, imaging was performed on a widefield microscopy equipped with 40× NA 0.75 PlanApo air immersion objective, an LED source (SPECTRA 4), an sCMOS camera (ZYLA 4.2 MP Plus), and a stage incubator (Tokai Hit, STRF-WELSXSET). If the repression of rRNA synthesis was not significant, imaging was performed again to confirm the effects at 24 h after drug treatment. All the candidate drugs were repeated twice.

### qRT-PCR

To examine the efficiency of shRNA-mediated knockdown or the changes of pre-rRNA expression, corresponding cells were collected using trypsin (Genecell). Total RNA was then extracted using FastPure Cell/Tissue Total RNA Isolation Kit (Vazyme) following the manufacturer’s instructions. RNA was converted to cDNA using oligo-dT primers and random hexamers (HiScript Ⅲ 1st Strand cDNA Synthesis Kit, Vazyme). PCR reactions were prepared using ChamQ Universal SYBR qPCR Master mix (Vazyme) and were performed on the CFX96 Real-Time PCR system (Bio-Rad). All reactions were done at least in triplicate. RNA abundance was normalized to an endogenous reference gene UBC and calculated as delta-delta threshold cycle (ΔΔCt). Primers used for qRT-PCR of UBC, UBF, SRFBP1, RRN3, and 45S pre-rRNA (Du et al., 2021) were listed in Table S6.

### Widefield microscopy

All widefield microscopy images were performed on a Nikon Ti2-E fluorescence microscope equipped with 40× NA 0.75/10× NA 0.30 PlanApo air immersion objectives, 100× NA 1.45 PlanApo oil immersion objective, an LED source (SPECTRA 4), an sCMOS camera (ZYLA 4.2 MP Plus), a Perfect Focus Unit (Nikon), and a motorized stage (Nikon) with stage incubator (Tokai Hit, STRF-WELSXSET). Cells were grown in 8-well chambered coverglass for imaging. To perform live-cell imaging, cells were maintained at 37°C and 5% CO_2_ in a humidified chamber. The following images were acquired on the widefield microscope: Figs. 3 G, 7 A, and 10 C. Other fluorescent images were acquired using confocal microscopy.

### Confocal microscopy

All confocal images were acquired on an Olympus IX83 fluorescence microscope equipped with spinning-disk confocal scanner (Yokogawa CSU-W1), a 60× NA 1.49 oil Apochromat objective, an sCMOS camera (Prime 95B), 405/488/561/640 nm lasers (OBIS), and a PIEZO stage (ASI) with stage incubator (Tokai Hit). For live-cell imaging, cells were maintained at 37°C and 5% CO_2_ in a humidified chamber. Cells for confocal imaging were plated into 8-well chambered coverglass. All supplementary movies were taken on the spinning-disk confocal microscope. *Z* stack images were processed by the projection of maximum intensity to generate the movies. Real-time imaging was recorded for 8 h with 4-min intervals to characterize transcriptional bursting of rDNA. Other imaging conditions were described in the legend of corresponding figures.

### Hessian-SIM imaging

Super-resolution imaging of nucleolar structures was performed using commercialized Hessian-SIM, termed HIS-SIM (High Intelligent and Sensitive Microscope) provided by Guang zhou Computational Super-resolution Biotech Co., Ltd. Images were acquired using a 100×/1.5 NA oil immersion objective (Olympus). Cells were seeded in 8-well chambered coverglass and maintained at 37°C and 5% CO_2_ in a humidified chamber for live SIM imaging. SIM images were collected and analyzed as described previously (Huang et al., 2018). Sparse deconvolution was carried out to further improve the image quality (Zhao et al., 2022).

### Data analysis

All the fluorescence imaging data were analyzed by ImageJ to calculate the mean intensity, total intensity, and the area of rRNA foci and NPM1 puncta. The area of rRNA foci was defined by the size of visible stdMCP-tdTomato spots, which was judged based on the signal intensity over background. A circular region of interest was drawn on each stdMCP-tdTomato dot to measure its area and mean signal intensity by ImageJ. Graph-Pad Prism (Version 5 and 8, GraphPad Software, https://www.graphpad.com) was used to calculate the mean/median values, statistical significance (defined as: * P < 0.05, ** P < 0.01, and *** P < 0.001), the SD and SEM. Line scan was performed using the “Analyze/Plot Profile” function, a plugin for ImageJ. The parameters were then analyzed in Excel and plotted in GraphPad Prism.

### Online supplemental material

Fig. S1 shows rRNA labeling through tagging various regions in rRNA. Fig. S2 shows the validation of CRISPR-based MS2 knockin in clonal cells by DNA sequencing. Fig. S3 shows the estimation of MS2 copy number in the genomic DNA of LiveArt clonal cells. Fig. S4 shows the characterizations of two additional 3′-ETS clones. Fig. S5 shows the detection of MS2-tagged rRNAs in ribosomes and DNA breaks/apoptosis in LiveArt clonal cells. Table S1 is a list of sgRNAs used in this study. Table S2 is a list of shRNAs used in this study. Table S3 is the DNA sequence of HITI donor for MS2 knockin. Table S4 is the DNA sequence of HDR donor for MS2 knockin. Table S5 is a list of qRT-PCR primers for ChIP assay. Table S6 is a list of qRT-PCR primers for detecting RNA abundance or copy number of MS2 cassette. Video 1 is a time-lapse confocal fluorescence video showing the validation of ITS1 rRNA labeling in clonal cells. Video 2 is a time-lapse confocal fluorescence video showing dynamic changes of rRNA synthesis throughout mitosis. Video 3 is a time-lapse confocal fluorescence video showing dynamics of nucleolar reassembly during mitosis in the absence of Actinomycin D. Video 4 is a time-lapse confocal fluorescence video showing dynamics of nucleolar reassembly during mitosis in the presence of Actinomycin D. Video 5 is a time-lapse confocal fluorescence video showing dynamics of the nucleolar structure in interphase. Video 6 is a time-lapse confocal fluorescence video showing dynamics of the nucleolar structure in interphase upon ActD treatment. Video 7 is a time-lapse confocal fluorescence video showing the transcriptional bursting of MS2-tagged 3′-ETS rRNA in interphase.

## Supplementary Material

Table S1 lists sgRNAs used in this study.

Table S2 lists shRNAs used in this study.

Table S3 lists the DNA sequence of HITI donor for MS2 knockin.

Table S4 lists the DNA sequence of HDR donor for MS2 knockin.

Table S5 lists qRT-PCR primers for ChIP assay.

Table S6 lists qRT-PCR primers for detecting RNA abundance or copy number of MS2 cassette.

SourceData F3 contains original blots for Fig. 3.

SourceData FS2 contains original blots for Fig. 2.

SourceData FS5 contains original blots for Fig. 5.
