# Supplementary material for: Real-time imaging of RNA polymerase I activity in living human cells
Source: J Cell Biol. 2022 Oct 25;222(1):e202202110. doi: 10.1083/jcb.202202110 (PMC9606689; doi:10.1083/jcb.202202110)
Supplement: Table S1 — lists sgRNAs used in this study. [file JCB_202202110_TableS1.docx]

**Table S1. sgRNAs used in this study**

| **sgRNA name** | **Target sequence** | | **PAM** |
| --- | --- | --- | --- |
| sg5’-ETS  sg18S  sgITS1  sg5.8S  sgITS2  sg28S  sg3’-ETS  sgTS3  sgrDNA-1  sgrDNA-2  sgrDNA-3  sgrDNA-4 | | GACACGCACGGCACGGAGCCAGC  GACCCGGGGAGGTAGTGAC  CGGGTGGGGGCTTTACCCGG  CGACACTTCGAACGCACTTG  CGTCCCGAGCTTCCGCGTCG  CGGCCGAGGTGGGATCCCG  GGAGCGTGGTTTGGGAGCCG  GCACCGATGCTCTCCGAGG  GGACGGCTGGGAAGGCCCGG  CCGGCCGAGGTGGGATCCCG  ACTCTGGTGGAGGTCCGTAG  TGATATAGACAGCAGGACGG | GGG  GGG  CGG  CGG  GGG  AGG  CGG  AGG  CGG  AGG  CGG  TGG |

Note: sgrDNA-1, -2, -3, -4 ( marked by green) were used for rDNA labeling using CRISPR imaging. Other sgRNAs were used for transient or stable labeling of rRNAs.
