# Supplementary material for: Real-time imaging of RNA polymerase I activity in living human cells
Source: J Cell Biol. 2022 Oct 25;222(1):e202202110. doi: 10.1083/jcb.202202110 (PMC9606689; doi:10.1083/jcb.202202110)
Supplement: Table S2 — lists shRNAs used in this study. [file JCB_202202110_TableS2.docx]

**Table S2. shRNAs used in this study**

| **Target gene** | **Target sequence (5’ to 3’)** |
| --- | --- |
| UBF  RRN3  SRFBP1  SRFBP1  SRFBP1 | GTACATTGACAGAATTGATCC  GAAGATGATGACTTTCTGAAA  GAAGTTGAGTCATCAAAGAAT  CCTCAGATCAAGAATCAGTTT  CCAGATTCTACTGCAACTGAA |
