# Supplementary material for: Real-time imaging of RNA polymerase I activity in living human cells
Source: J Cell Biol. 2022 Oct 25;222(1):e202202110. doi: 10.1083/jcb.202202110 (PMC9606689; doi:10.1083/jcb.202202110)
Supplement: Table S3 — lists the DNA sequence of HITI donor for MS2 knockin. [file JCB_202202110_TableS3.docx]

**Table S3. The DNA sequence of HITI donor for MS2 knockin**

Sequence (5' to 3')

GCACCGATGCTCTCCGAGG**AGG**GAAGGTGACAACCGGTAACCTACAAACGGGTGGAGGATCACCCCACCCGACACTTCACAATCAAGGGGTACAATACACAAGGGTGGAGGAACACCCCACCCTCCAGACACATTACACAGAAATCCAATCAAACAGAAGCACCATCAGGGCTTCTGCTACCAAATTTATCTCAAAAAACTACAACAAGGAATCACCATCAGGGATTCCCTGTGCAATATACGTCAAACGAGGGCCACGACGGGAGGACGATCACGCCTCCCGAATATCGGCATGTCTGGCTTTCGAATTCAGTGCGTGGAGCATCAGCCCACGCAGCCAATCAGAGTCGAATACAAGTCGACTTTCGCGAAGAGCATCAGCCTTCGCGCCATTCTTACACAAACCACACTCTCCCCTACAGGAACAGCATCAGCGTTCCTGCCCAGTACCCAACTCAAGAAAATTTATGTCCCCATGCAGCATCAGCGCATGGGCCCCAAGAATACATCCCCAACAAAATCACATCCGAGCACCAACAGGGCTCGGAGTGTTGTTTCTTGTCCAACTGGACAAACCCTCCATGGACCATCAGGCCATGGACTCTCACCAACAAGACAAAAACTACTCTTCTCGAAGCAGCATCAGCGCTTCGAAACACTCGAGCATACATTGTGCCTATTTCTTGGGTGGACGATCACGCCACCCATGCTCTCACGAATTTCAAAACACGGACAAGGACGAGCACCACCAGGGCTCGTCGTTCCACGTCCAATACGATTACTTACCTTTCGGGATCACGATCACGGATCCCGCAGCTACATCACTTCCACTCAGGACATTCAAGCATGCACGATCACGGCATGCTCCACAAGTCTCAACCACAGAAACTACCAAATGGGTTCAGCACCAGCGAACCCACTCCTACCTCAAACCTCTTCCGCACCGATGCTCTCCGAGG**AGG**

(Text marked by yellow is the 17 copies of MS2V5; green text is the TS3 sequence which can be cleaved by Cas9. The underlined bold text is the PAM sequence of TS3.)
