# Supplementary material for: Real-time imaging of RNA polymerase I activity in living human cells
Source: J Cell Biol. 2022 Oct 25;222(1):e202202110. doi: 10.1083/jcb.202202110 (PMC9606689; doi:10.1083/jcb.202202110)
Supplement: Table S5 — lists qRT-PCR primers for ChIP assay. [file JCB_202202110_TableS5.docx]

**Table S5. qRT-PCR primers for ChIP assay**

| **Primer name** | **5’-oligo** | **3’-oligo** |
| --- | --- | --- |
| H0.1  H1  H4  H8  H27  H42.8 | CTGACACGCTGTCCTCTG  GGCGGTTTGAGTGAGACGAGA  CGACGACCCATTCGAACGTCT  AGTCGGGTTGCTTGGGAATGC  CCTTCCACGAGAGTGAGAAGCG  GTGTGTGGCTGCGATGGT | AGGCGGCTCAAGGCAGGA  ACGTGCGCTCACCGAGAGCAG  CTCTCCGGAATCGAACCCTGA  CCCTTACGGTACTTGTTGACT  CTCGACCTCCCGAAATCGTACA  CTCGGAGCGAAAGATATACCTCCC |
