# Supplementary material for: Real-time imaging of RNA polymerase I activity in living human cells
Source: J Cell Biol. 2022 Oct 25;222(1):e202202110. doi: 10.1083/jcb.202202110 (PMC9606689; doi:10.1083/jcb.202202110)
Supplement: Table S6 — lists qRT-PCR primers for detecting RNA abundance or copy number of MS2 cassette. [file JCB_202202110_TableS6.docx]

**Table S6. qRT-PCR primers for detecting RNA abundance or copy number of MS2 cassette**

| **Gene of interest** | **5’-oligo** | **3’-oligo** |
| --- | --- | --- |
| 45S pre-rRNA  UBF  RRN3  SRFBP1  *MUC4*  MS2V5_17X_ | GCCTTCTCTAGCGATCTGAGAG  GGCCAAGTATGCGAAACTCC  ATGACCAGATCATCAACTGGC  CTCAGCCGGGAACTCTGAAC  GAACCACAGAGGACACATTAAT  CGATTACTTACCTTTCGGGATC | CCATAACGGAGGCAGAGACA  GTTTCGCTCGAACTCCTGTT  GGAAAACAGTCTGTGCTGATACA  ACAGTGCATCTTCAGTACCC  CTGAGATGAAGCTGATATGTCCT  GCTGAACCCATTTGGTAGTTTC |
