# Supplementary figures and images for: Real-time imaging of RNA polymerase I activity in living human cells
Source: J Cell Biol. 2022 Oct 25;222(1):e202202110. doi: 10.1083/jcb.202202110 (PMC9606689; doi:10.1083/jcb.202202110)

D

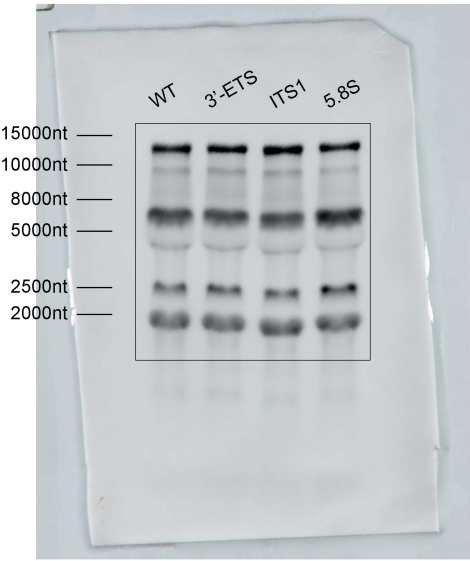

probe: hITS1  
sequence: 5'-AAGGGGTCTTTAAACCTCCGCGCC-3'

E

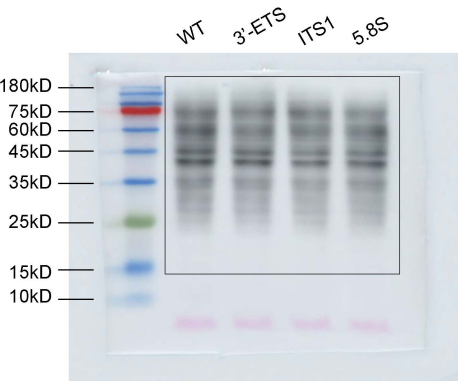

Puromycin

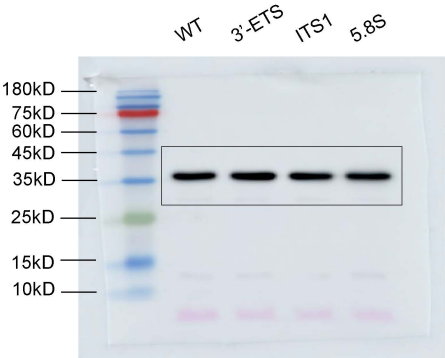

GAPDH

Supplement: SourceData F3 — contains original blots for Fig. 3. [file JCB_202202110_SourceDataF3.pdf]

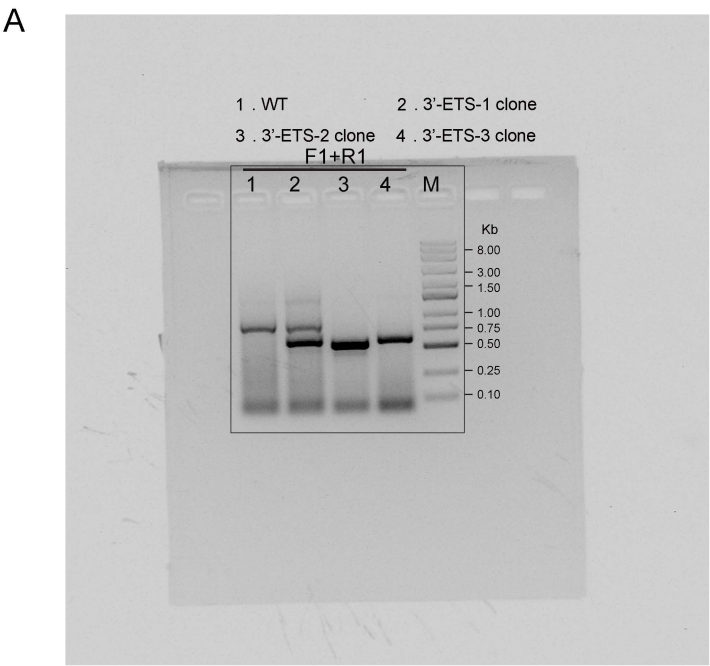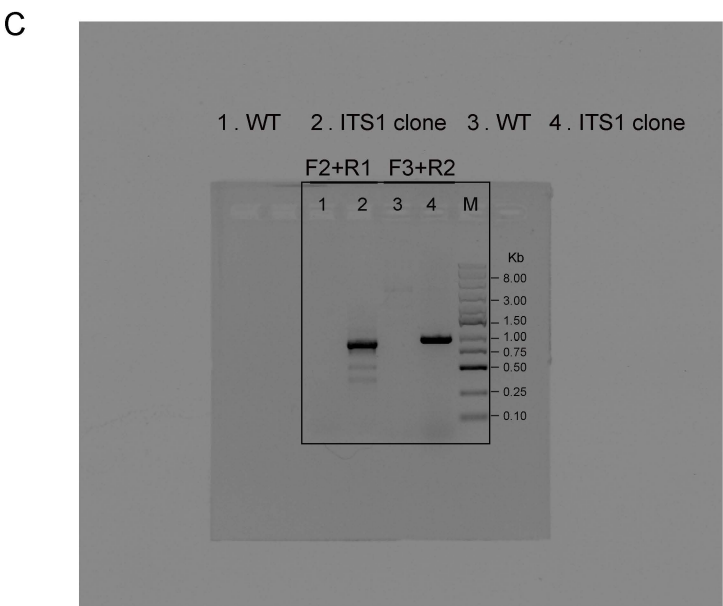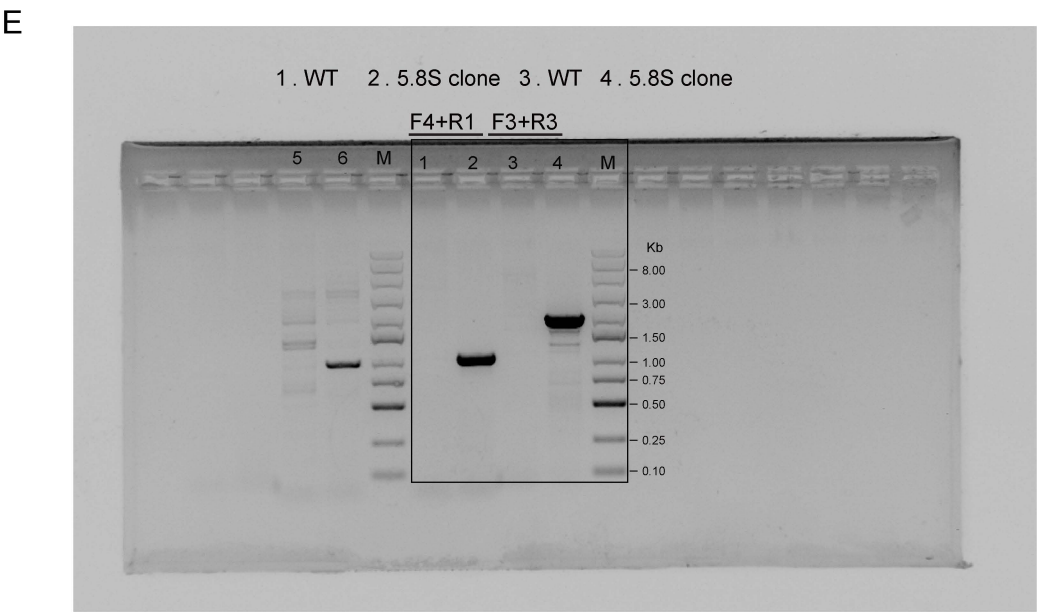

The two bands on the left (5, 6) are unrelated to this experiment.

Supplement: SourceData FS2 — contains original blots for Fig. 2. [file JCB_202202110_SourceDataFS2.pdf]
