# Supplementary material for: Real-time imaging of RNA polymerase I activity in living human cells
Source: J Cell Biol. 2022 Oct 25;222(1):e202202110. doi: 10.1083/jcb.202202110 (PMC9606689; doi:10.1083/jcb.202202110)
Supplement: SourceData FS5 — contains original blots for Fig. 5. [file JCB_202202110_SourceDataFS5.pdf]

E

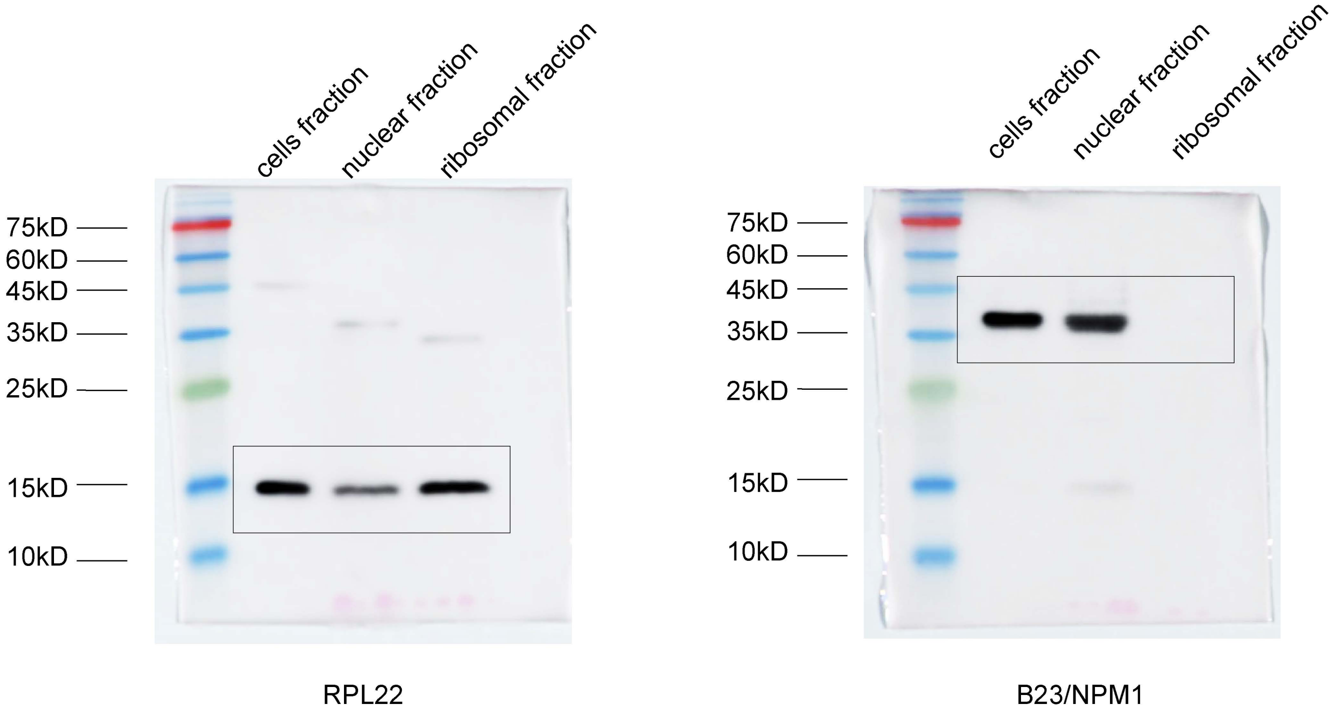

F

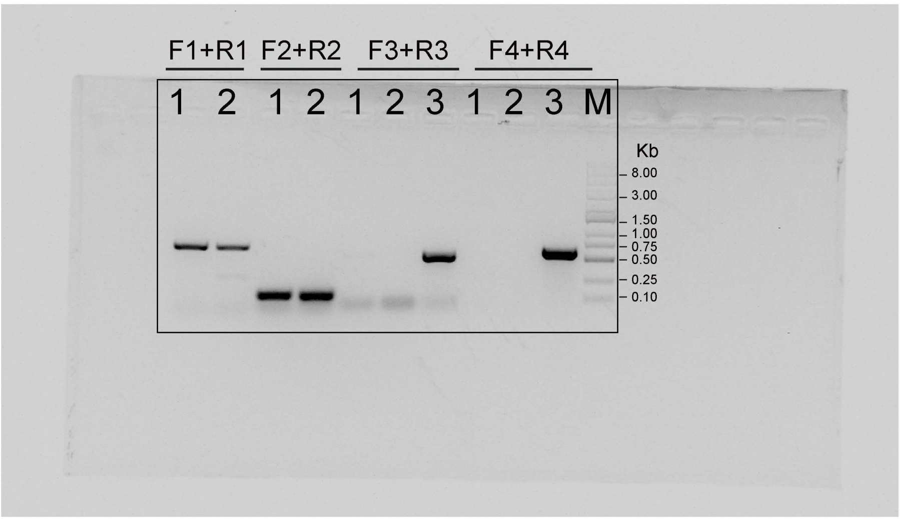

PCR templates

1. Ribosomal cDNA of WT cells
2. Ribosomal cDNA of 5.8S clone
3. Genomic DNA of 5.8S clone
